# Immunometabolic Signatures Predict Risk of Progression to Active Tuberculosis and Disease Outcome

**DOI:** 10.3389/fimmu.2019.00527

**Published:** 2019-03-22

**Authors:** Fergal J. Duffy, January Weiner, Scott Hansen, David L. Tabb, Sara Suliman, Ethan Thompson, Jeroen Maertzdorf, Smitha Shankar, Gerard Tromp, Shreemanta Parida, Drew Dover, Michael K. Axthelm, Jayne S. Sutherland, Hazel M. Dockrell, Tom H. M. Ottenhoff, Thomas J. Scriba, Louis J. Picker, Gerhard Walzl, Stefan H. E. Kaufmann, Daniel E. Zak, The GC6-74 Consortium, S. H. E. Kaufmann

**Affiliations:** Author Affiliations: Germany: GC6-74 Principal Investigator; Department of Immunology, Max Planck Institute for Infection Biology, Berlin; South Africa: DST/NRF Centre of Excellence for Biomedical TB Research and MRC Centre for TB Research, Division of Molecular Biology and Human Genetics, Stellenbosch University, Tygerberg; South African Tuberculosis Vaccine Initiative, Institute of Infectious Disease and Molecular Medicine & Division of Immunology, Department of Pathology, University of Cape Town, Cape Town; Netherlands: Department of Infectious Diseases, Leiden University Medical Centre, Leiden; University Medical Centre, Utrecht; USA: Tuberculosis Research Unit, Department of Medicine, Case Western Reserve University School of Medicine and University Hospitals Case Medical Center, Cleveland, Ohio; Aeras, Rockville, MD; Uganda: Department of Microbiology and Immunology, Stanford University, Stanford, California; Uganda: Department of Medicine and Department of Microbiology, College of Health Sciences, Faculty of Medicine, Makerere University, Kampala; UK: Department of Immunology and Infection, Faculty of Infectious and Tropical Diseases, London School of Hygiene & Tropical Medicine, London; Malawi: Karonga Prevention Study, Chilumba; Ethiopia: Ethiopian Health & Nutrition Research Institute, Addis Ababa; Armauer Hansen Research Institute, Addis Ababa; The Gambia: Vaccines &Immunity Theme, Medical Research Council Unit, Fajara; Denmark: Department of Infectious Disease Immunology, Statens Serum Institute, Copenhagen.; ^1^Center for Global Infectious Disease Research, Seattle Childrens Research Institute, Seattle, WA, United States; ^2^Max Planck Institute for Infection Biology, Berlin, Germany; ^3^Oregon Health and Science University, Portland, OR, United States; ^4^Division of Molecular Biology and Human Genetics, Department of Biomedical Sciences, SAMRC-SHIP South African Tuberculosis Bioinformatics Initiative (SATBBI), Center for Bioinformatics and Computational Biology, DST/NRF Centre of Excellence for Biomedical Tuberculosis Research, South African Medical Research Council Centre for Tuberculosis Research, Stellenbosch University, Stellenbosch, South Africa; ^5^Department of Pathology, South African Tuberculosis Vaccine Initiative, Institute of Infectious Disease and Molecular Medicine & Division of Immunology, University of Cape Town, Cape Town, South Africa; ^6^Center for Infectious Disease Research, Seattle, WA, United States; ^7^Translational Medicine & Global Health Consulting, Berlin, Germany; ^8^Vaccines & Immunity Theme, Medical Research Council Unit, Fajara, Gambia; ^9^Department of Immunology and Infection, London School of Hygiene and Tropical Medicine, London, United Kingdom; ^10^Department of Infectious Diseases, Leiden University Medical Center, Leiden, Netherlands

**Keywords:** rhesus macaque, household contact, biomarker, transcriptomics, metabolomics, tuberculosis, inflammation, host-pathogen interaction

## Abstract

There remains a pressing need for biomarkers that can predict who will progress to active tuberculosis (TB) after exposure to Mycobacterium tuberculosis (MTB) bacterium. By analyzing cohorts of household contacts of TB index cases (HHCs) and a stringent non-human primate (NHP) challenge model, we evaluated whether integration of blood transcriptional profiling with serum metabolomic profiling can provide new understanding of disease processes and enable improved prediction of TB progression. Compared to either alone, the combined application of pre-existing transcriptome- and metabolome-based signatures more accurately predicted TB progression in the HHC cohorts and more accurately predicted disease severity in the NHPs. Pathway and data-driven correlation analyses of the integrated transcriptional and metabolomic datasets further identified novel immunometabolomic signatures significantly associated with TB progression in HHCs and NHPs, implicating cortisol, tryptophan, glutathione, and tRNA acylation networks. These results demonstrate the power of multi-omics analysis to provide new insights into complex disease processes.

## Introduction

Tuberculosis (TB) is an infectious disease caused by the bacterial pathogen *Mycobacterium tuberculosis (M. tb)*, which is spread via aerosolized droplets that originate from the expectorations of diseased individuals. In 2017, it was estimated that 10 million people fell ill with TB and 1.6 million died from the disease. Overall, about 10% of individuals latently infected with *M.tb* will progress to active disease at some point in their lives (1). However, the risk of progression is higher for certain groups, including HIV+ individuals, children (2–4), and those with metabolic and nutritional conditions such as diabetes (5, 6) and Vitamin A deficiency (7). Progression is also more frequent immediately post-contact with a TB patient: sharing a household with an active TB case is associated with elevated risk of exposure to *M.tb* and subsequent development of active disease (8–12). Major obstacles to fighting TB are the lack of effective TB diagnostics and the extremely large number of latently infected individuals, estimated at 23% of the world's population (13). Due to the impracticality of effectively treating all latently infected individuals and the accompanying possible side effects of such treatments, an effective method for identifying individuals at high risk of progression to active TB disease is highly desirable. Since *M. tb* is spread by individuals with active TB, early identification and treatment of high-risk individuals could break the chain of transmission and facilitate control of the TB epidemic.

A deeper understanding of immune dysregulation that leads to active TB disease also has the potential to point the way toward novel interventions to prevent progression. Blood transcriptional profiles offer the advantage of being easily monitored and strongly indicative of immune perturbations driven by TB disease. Previous studies have identified transcriptional signatures in peripheral blood that discriminate active TB from latent TB (14–23). In addition to transcriptional approaches, the development of sensitive metabolic profiling technologies has allowed investigation of relationships between specific metabolites and immune functions (24). Metabolomic profiling can detect non-transcriptional changes in cellular activity as well as metabolites released into the plasma from local tissue sites. Metabolomics has been used to develop specific signatures of TB disease which implicated inflammatory and hypoxic metabolic pathways (25, 26). Recently, integration of transcriptomic and metabolomic measurements in healthy individuals was shown to reveal systematic relationships between serum metabolite and blood transcript levels in various signaling, transport, and metabolic processes (27). By taking a similar approach within the context of TB, immunometabolic processes that are altered during TB progression may be revealed.

This study uses RNAseq and metabolomic profiling of household contacts of TB index cases (HHC) samples that were collected as part of the Bill and Melinda Gates Grand Challenges 6–74 program (GC6–74). These cohorts have previously been used to successfully validate a transcriptional signature of TB risk (28). Furthermore, RNAseq (29), metabolomic profiling (30), and c-miRNA (31) analyses of these cohorts identified and validated novel transcriptional and metabolomic signatures of risk for TB. We hypothesized that the ability to predict and understand the processes underlying TB progression after exposure to TB will be improved by integrating the transcriptional and metabolomic profiles for the GC6–74 HHC cohorts. We demonstrate this improvement through the realization of increased prediction accuracy when applying existing transcriptional and metabolic signatures of TB risk and disease and through the *de-novo* identification of TB progression-associated functional immunometabolomic pathway elements. Furthermore, using whole blood RNA and plasma metabolomic profiles measured 28 days after challenge, we independently validate the multi-omic signatures by applying them to predict the spectrum of TB-associated disease (measured at necropsy) observed in rhesus macaques (RMs) that had been vaccinated with the TB vaccine candidate RhCMV/MTB, named according to its design as rhesus cytomegalovirus vector (RhCMV) encoding *M.tb* antigen repeats. Partial protection has been observed after vaccination with RhCMV/MTB, allowing evaluation of correlates of TB risk (32).

## Results

### Multi-Omics Analysis Strategy to Identify and Validate Immunometabolic Signatures for Risk of Progression to Active Tuberculosis

By employing a multi-step analytical strategy (Figure S1), we tested whether integration of blood transcriptional profiling with serum metabolomic profiling can provide new understanding of disease processes and enable improved prediction of TB progression. As detailed below, this analysis involved combined testing of a pre-existing transcriptional TB risk signature (28) and a metabolic TB disease signature (25) on the GC6-74 HHC cohort (28) and a stringent non-human primate (NHP) TB challenge model (32), followed by direct integrated analysis of GC6-74 RNAseq (29) and metabolomics (30).

### Household Contact Study Design, Participant Recruitment, and Sample Processing

The GC6-74 cohort study design has been previously described (28). GC6-74 comprised HIV-negative household contacts of active TB cases that were recruited from four African study sites, in South Africa (Stellenbosch University/SUN), The Gambia (Medical Research Council Unit The Gambia/MRC), Uganda (Makerere University/MAK), and Ethiopia (Armauer Hansen Research Institute/AHRI). Whole blood samples were taken from study participants at enrollment of HHCs (BL: which was within 2 months of the exposure event). Where feasible, further whole blood samples were taken 6 months post-enrollment (M6) and 18 months post enrollment (M18), provided the individual remained free of active TB.

Study participants that developed microbiologically confirmed active TB between up to 2 years after HHC were termed TB “progressors” and participants who remained TB free after HHC were termed “controls.” Progressors that developed TB within 3 months of HHC were excluded from further analysis as possible co-incident cases. Controls were matched to progressors by age, sex, study site, and year of enrollment (Table 1). Mass-spectrometry based metabolomic profiling of collected blood-derived plasma (The Gambia, Ethiopia) and serum (South Africa, Uganda) along with RNA sequencing (RNAseq) of whole blood total RNA was performed for available progressor and control samples. Progressor and control samples for which both RNAseq and metabolomic profiling were successfully completed were analyzed in the present study. The total number of metabolomic and RNAseq transcriptional profiles available for each site is shown in Table 1. This shared dataset was dominated by South African and Gambian donors, with a smaller number of samples from Ethiopian donors, and a single Ugandan control sample.

**Table 1:** Sample Counts by cohort, TB progression status, and sample type.

		**RNAseq**	**Metabolomics**	**Shared RNAseq and Metabolomics**
South Africa (SUN)	Progressor	43	81	40
	Control	153	255	134
The Gambia (MRC)	Progressor	39	61	36
	Control	130	190	121
Ethiopia (AHRI)	Progressor	16	20	15
	Control	32	59	19
Uganda (MAK)	Progressor	1	19	1
	Control	2	66	0

*Cohorts are labeled by the short form of their recruitment institution: SUN (Stellenbosch University, South Africa), MRC (Medical Research Council, the Gambia), AHRI (Armaeur Hanser Research Institute, Ethiopia), and MAK (Makerere University, Uganda)*.

### RhCMV-Vaccinated Rhesus-Macaque Study Design and Sample Processing

Transcriptional and metabolomic profiles were obtained from rhesus macaques (RMs) vaccinated with cytomegalovirus-vectors encoding M.tb antigen inserts (RhCMV/TB) prior to *M.tb* challenge (32). These samples were obtained from two independent RM challenge groups, comprising a total of 59 RMs. Blood was drawn 4 weeks post-challenge with RNAseq and metabolomic profiling being performed as for the human samples. Disease outcome was measured as the harmonized disease score at necropsy, performed either upon clinical diagnosis of active TB (10–20 weeks post challenge), or in a randomized manner 16–30 weeks post challenge in the absence of a positive TB diagnosis. The harmonized disease score was identical to that reported in the original publication, representing a scaled summary of lung pathology and TB culture growth from tissues collected at necropsy (32).

### Combining Existing Transcriptomic and Metabolomic Signatures Significantly Improves Blind Prediction of TB Progression in GC6-74 HHCs

As a first step to evaluate whether combined transcriptional and metabolomic analysis of the HHC cohort would yield more accurate prediction of TB progression, we used transcriptional and metabolomic signatures developed from independent study cohorts. This approach allows the use of all GC6-74 samples as an independent test set, increasing statistical power. For the transcriptional signature, we employed the Adolescent Cohort Study Correlate of Risk (ACS-CoR). This previously-described signature of risk of TB progression is comprised of 63 splice junctions from 16 genes and was developed from whole blood RNAseq analysis of a cohort of latently-infected adolescents from a TB-endemic region of South Africa (28), [GEO: GSE94438]. For the metabolomic signature, we re-derived a 25-metabolite diagnostic signature, termed Metabolomics Active Disease Signature (MetabAD, Table S1). The signature was trained on data from a published dataset of metabolite profiles measured in the serum of South African adults and adolescents with active TB, latent infection, and healthy controls (25), with metabolites not also detected in all three GC6-74 sites being removed before model fitting.

**Figure 1:**
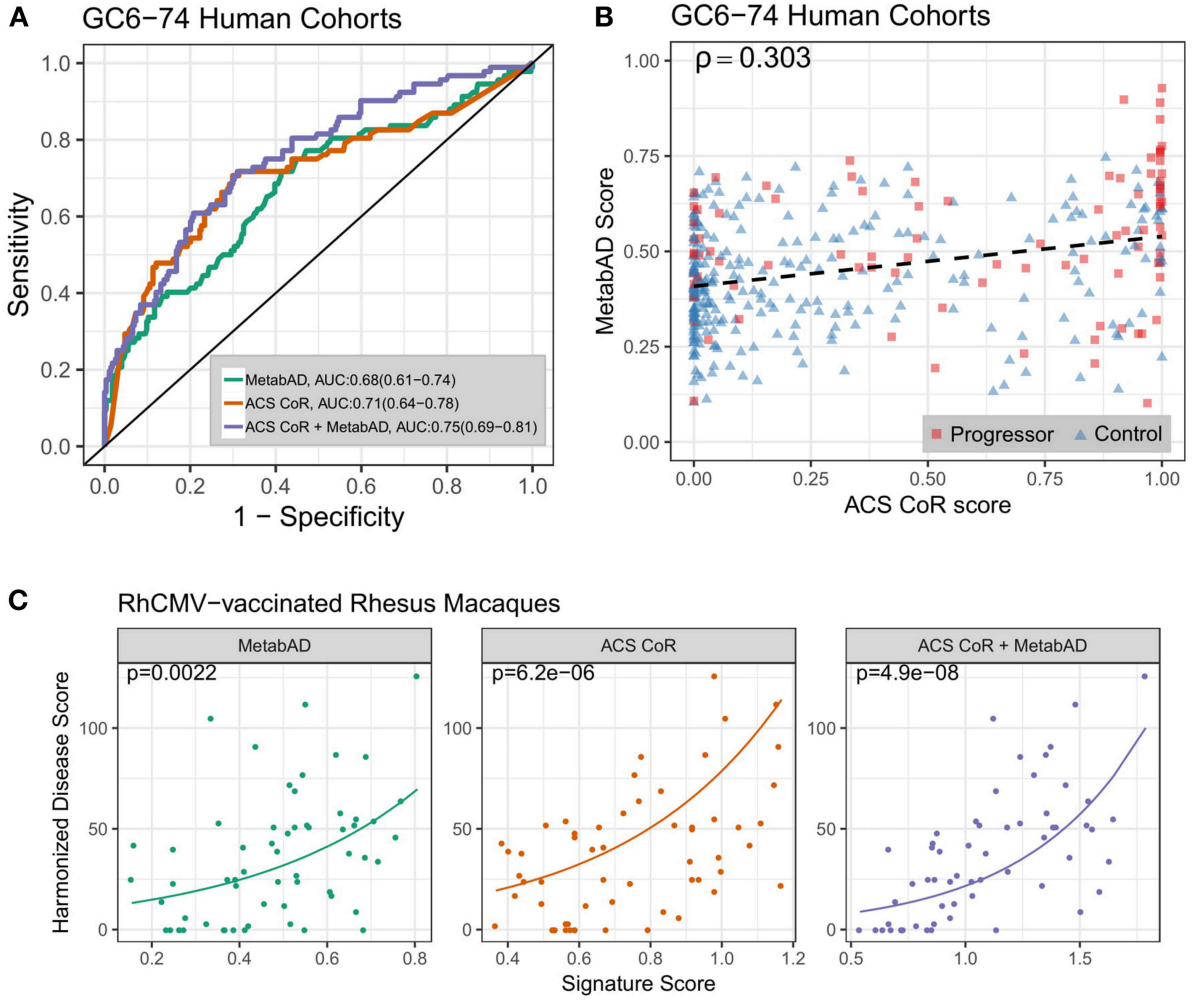
Performance of a combined transcriptomic and metabolomic signature of TB progression. **(A)** ROC curves for the ACS CoR transcriptomic signature alone, the MetabAD metabolomic signature alone, and the sum of ACS CoR + MetabAD. Legend shows signature AUCs and bootstrapped 95% confidence intervals around the AUC in parentheses. **(B)** Scatter plot of ACS CoR scores (x-axis) vs. MetabAD scores (y-axis). Progressor samples are shown as red squares, and Control samples are shown as blue triangles, with signature correlation indicated in the upper left (Spearman's ρ). The dashed black line indicates the linear fit of MetabAD vs. ACS CoR. **(C)** Scatter plots of individual and combined ACS CoR and MetabAD signature scores vs. harmonized disease score in two RhCMV-vaccinated rhesus macaque studies after *M.tb* challenge. Poisson regression was used to determine the relationship between signature score, measured 28 days post-challenge and harmonized disease score at time of necropsy. Solid lines represent Poisson regression fits to the harmonized disease score for MetabAD, ACS CoR and ACS CoR + MetabAD, respectively and *p*-values shown in the top left of each plot indicate significance of association between signature score and harmonized disease score.

We computed the ACS-CoR and MetabAD signature scores for the GC6-74 samples for which both RNAseq and metabolomics measurements were available (Table 1). As previously-reported (28), ACS CoR significantly discriminated TB progressors from controls amongst GC6-74 HHCs [ROC AUC = 0.71 (95% CI:0.64–0.78)]. Although derived from active disease datasets, the diagnostic MetabAD also significantly discriminated GC6-74 HHC progressors from controls [ROC AUC = 0.68 (95% CI 0.61–0.74); Figure 1A]. Binary classification of GC6-74 samples by both signatures using optimal discrimination thresholds indicated that, of 366 samples, 177 were correctly classified by both signatures, 80/336 samples were correctly classified by ACS CoR but incorrectly by MetabAD, and 50 were correctly classified by MetabAD but incorrectly classified by ACS CoR (Table S2). Despite these differences, the scores for the ACS-CoR and MetabAD signatures were significantly correlated, albeit weakly (Spearman's ρ = 0.30). As shown in Figure 1B, some TB progressor samples had high MetabAD scores and low ACS CoR scores, and vice-versa. Both signatures produce prediction scores in the range [0, 1], therefore scores from metabolomics and transcriptomic signatures lie on the same theoretical scale. To formally assess the prediction improvements attainable by applying the two signatures together, we computed a combined transcriptomics + metabolomics signature score for each sample by adding the two individual scores together. This sum represents a parameter-free combined signature score that did not require the fitting of an additional model to the data. This combined additive signature showed an AUC of 0.75 (0.69–0.81) (Figure 1A), a significant improvement over either signature alone (ACS-CoR χ^2^ *p* = 0.0006; or MetabAD χ^2^ *p* =7× 1 0^−8^) for all samples. The greatest prediction performance improvement was observed on samples within 6 months of TB diagnosis, with the combined model achieving ROC AUC of 0.8 (0.7–0.9) compared to ROC AUC of 0.7 (0.58–0.82) and 0.73 (0.58–0.89) for the individual MetabAD and ACS CoR models, respectively (Figure S2A). The combined signature also showed improved predictive performance vs. both ACS CoR and MetabAD independently for all three study sites (South Africa, The Gambia, Ethiopia, Figures S2B–D).

Further validation of this result was performed using transcriptional and metabolomic profiles measured 28 days post *M.tb* challenge in RMs from the RhCMV/TB vaccine study (32). This was performed by evaluating the association between the ACS CoR and MetabAD signature scores and the RM outcome harmonized disease score. As the harmonized disease score is a strictly positive number derived from count-based measures (i.e., necropsy score and number of positive necropsy cultures), Poisson modeling was used to evaluate the association of signature scores with harmonized disease scores. Figure 1C illustrates the prospective association of ACS-CoR, MetabAD and the sum of ACS-CoR + MetabAD with the harmonized disease score. The combination of ACS-CoR with MetabAD shows a significantly stronger association with disease outcome (*p* = 4.9 × 10^−8^, Kendall's τ = 0.5) than either ACS-CoR (*p* = 6.2 × 10^−6^, τ = 0.34) or MetabAD (*p* = 0.0022, τ = 0.31) alone.

Altogether, these results demonstrate that the pre-defined transcriptomic and metabolomic signatures each capture complementary TB-related biological variation that is not present in the other signature. Combining them yields a significantly improved signature of risk for TB that also prospectively captures the spectrum of post-challenge disease severity in RhCMV/MTB-vaccinated RM.

### The Transcriptome and Metabolome Provide Complementary Information on TB Progression

Given that the specific case of ACS-CoR and MetabAD signatures demonstrated the benefit of combining transcriptomic and metabolomic measurements for predicting TB progression, we sought to more globally quantify the benefit of combining data from transcriptional and metabolomic platforms. Specifically, we determined whether combining an individual transcript with an individual metabolite more frequently resulted in a significant improvement in prediction performance than combining two transcripts or two metabolites. After performing an initial filtering step to remove transcripts and metabolites lacking any univariate association with TB progression (*t*-test *p* > 0.05), pairwise logistic regression models fitting TB progression as a function of all possible *transcript-transcript (t-t), metabolite-metabolite (m-m)*, and *transcript-metabolite (t-m)* pairs from the remaining 5,892 transcripts and 195 metabolites were constructed. Each pairwise model was tested for significant complementarity between the two features, which was defined as a significant improvement (χ^2^ *p* < 0.05) for the pair model compared to univariate logistic regression models for either individual element alone. All resulting *(t-t), (t-m)*, and *(m-m)* pairs that exhibited complementarity between the features are listed in Table S3. While the majority (64%) of *(t-m)* pairings exhibited complementarity, this was observed for only a minority of *(t-t)* pairs (18%) and *(m-m)* pairs (42%) (Table 2). This result indicates that, in a general sense, transcriptional and metabolomic measurements provide non-redundant information that is relevant for predicting risk of TB progression.

**Table 2:** Proportion of transcript-metabolite, transcript-transcript, and metabolite-metabolite pairs that show significant complementarity in prediction of TB progression.

	**No significant improvement over either element alone**	**Significant improvement over both individual elements alone**
Transcript (*n* = 5,892)—Metabolite (*n* = 195) *(t-m)* pairings	**36%**	**64%**
Metabolite—Metabolite *(m-m)* pairings (*n* = 195 × 195)	58%	42%
Transcript—Transcript *(t-t)* pairings (*n* = 5,892 × 5,892)	82%	18%

*Bold values serve to highlight the most important metabolomic + transcriptomics row*.

### Correlations Between Transcripts and Metabolites Reveal Biological Networks Involved in TB Progression

**Figure 2:**
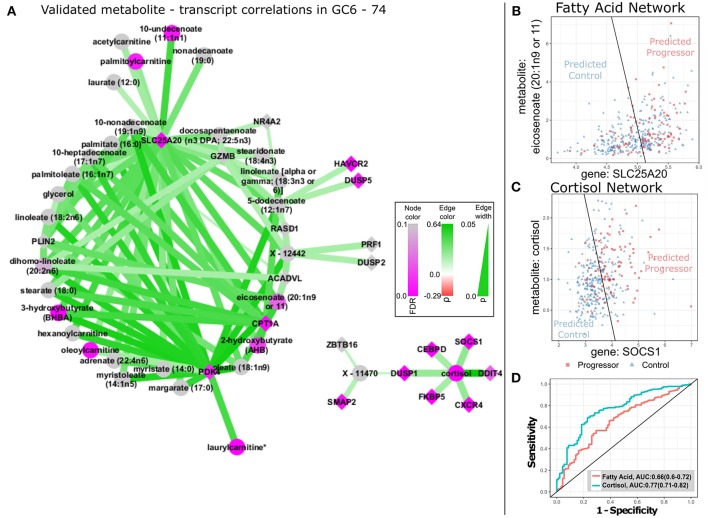
TB-related biological networks inferred from correlations in GC6-74. **(A)** Network plot of selected transcript/metabolite pairs previously identified as correlated in KORA F4 that are also significantly correlated in GC6-74 samples. Transcript nodes are shown as diamonds, metabolite nodes as circles, with significant correlations indicated by edges linking transcripts and metabolites. Positive correlations between metabolites and transcripts are shown as green edges and negative correlations as red. Darker shades indicate stronger correlations (legend at center right). Transcripts and metabolites that showed significant association with TB progression are shaded in purple, with unassociated nodes shaded gray. Darker shades indicate more significant association, according to legend in bottom left. **(B,C)** Scatter plots of the levels of the optimal fatty-acid (SLC25A20/eicosenoate) and cortisol (SOCS1/Cortisol) transcript (y-axis)/ metabolite (x-axis) pairs in all GC6-74 samples. Progressor samples are shown as red squares, and control samples are shown as blue triangles. The optimal logistic regression classification boundary for each pair is shown as a black line, and text labels “Predicted Progressor” and “Predicted Control” indicate logistic regression binary predictions either side of the classification boundary. **(D)** ROCs for the fatty-acid and cortisol logistic regression pair models shown in **(B,C)** predicting all GC6-74 samples. AUCs for each model are shown in the legend, with 95% CIs in parentheses.

We sought to explore biological relationships between transcript and metabolite abundances by comprehensively evaluating rank correlations between all detectable transcripts (*n* = 15,320) and metabolites (*n* = 830). A total of 11,851 statistically significant correlations were identified (FDR<0.05). We validated these significant (t-m) correlations by testing them on an independent set of transcriptional and metabolomic profiles measured in healthy elderly German adults from the KORA F4 cohort (27). Despite demographic differences between the GC6-74 and KORA F4 cohorts, (t-m) correlations between the studies were significantly concordant (*p* = 2.61 × 10^−88^). Furthermore, of 1,109 significant correlations identified in KORA F4, 181 were also significant in GC6-74 (FDR = 0.05), and correlation coefficients (Spearman's ρ) for the significant KORA F4 transcript-metabolite pairs were highly correlated between the studies (ρ = 0.59, *p* < 2 × 10^−16^) (Figures S3, S4). Thus, robust correlations between the abundance of particular transcripts in whole blood and particular metabolites in serum/plasma were consistently observed in two very different human cohorts, suggesting that these transcripts and metabolites are functionally linked. We next identified the subset of (t-m) pairs that were significantly correlated in both GC6-74 and KORA F4 and that were significantly impacted by TB progression (Figure 2A). The resulting data-driven immunometabolic network was dominated by a core of hub genes connected to multiple metabolites. Three hub genes are significantly associated with TB progression: the mitochondrial fatty-acid metabolism genes *CPT1A, SLC25A20*, and *PDK4*. Correlated with these genes are fatty acid metabolites and related molecules such as carnitines, some of which are also associated with TB progression. In order to estimate the potential of the fatty-acid metabolism network for predicting TB progression, logistic regression models were fitted as above for each of the (t-m) pairs in this network. The best fit pair model was *SLC25A20*:eicosenoate (20:1n9 or 11), with an AUC of 0.66 (0.60–0.72) (Figures 2B,D). Another data-driven immunometabolic subnetwork was centered around a correlation between cortisol and immune signaling genes such as *FKBP-5, CXCR4, CEBPD, DDIT4*, and *SOCS1*. This small subnetwork exhibits strong potential for predicting TB with an AUC of 0.77 (0.71–0.82) (Figures 2C,D).

### Joint Pathway Analysis of Transcripts and Metabolites Reveals Canonical Biochemical Pathways Altered in TB Progression

**Figure 3:**
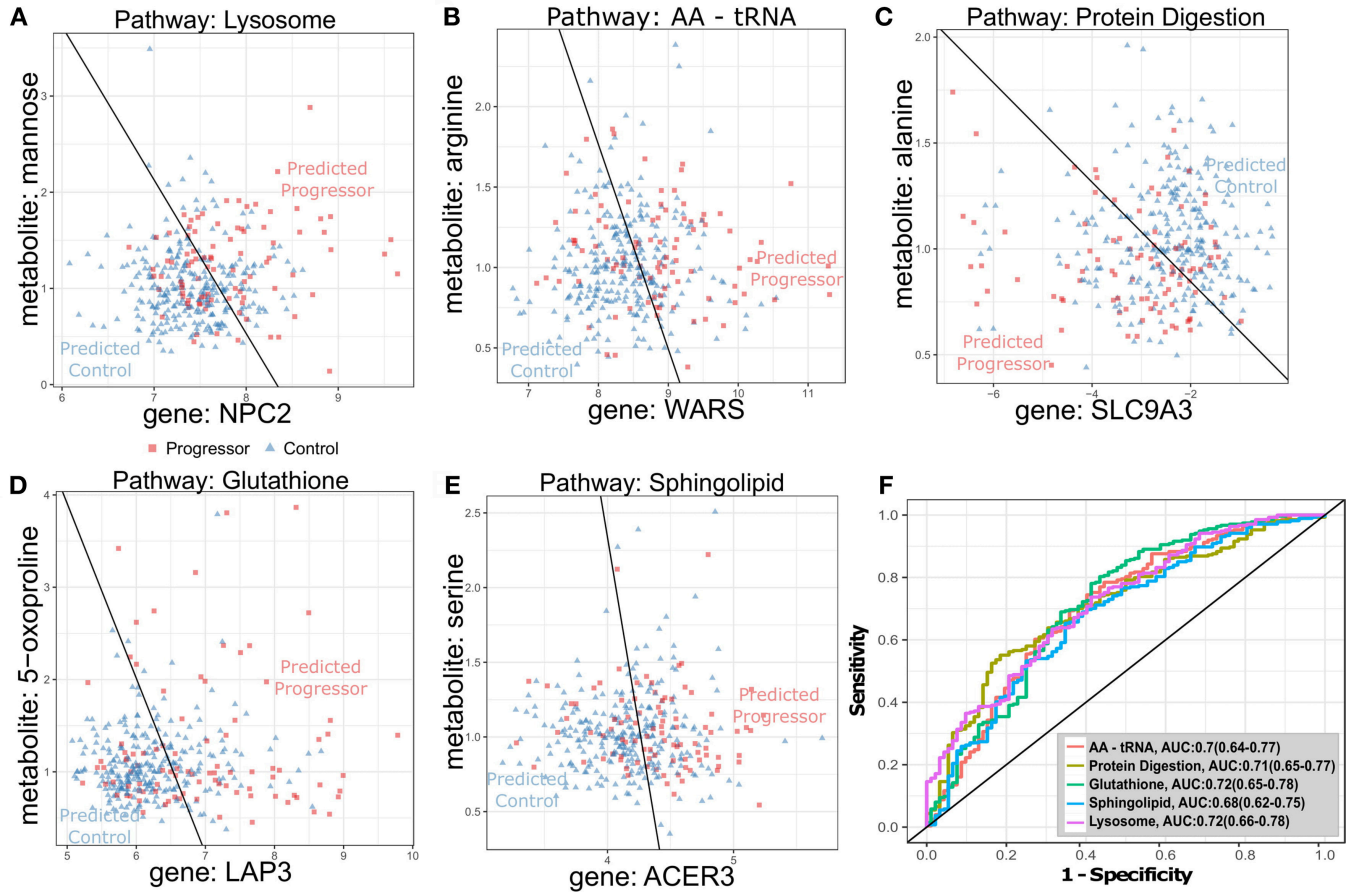
Optimal transcript-metabolite pairs derived from canonical pathways predictive of TB progression in GC6-74 **(A–E)**: Scatter plots of transcript (x-axis) and metabolite (y-axis) expression for transcript-metabolite pairs from canonical pathways significantly enriched for differential expression in all GC6-74 samples: Lysosome, AA-tRNA, protein digestion, glutathione and sphingolipid, respectively. Samples taken from TB progressors (Progressor) are shown as red squares, and samples from non-progressors (Control) are shown as blue triangles. The optimal logistic regression classification boundary is shown as a black line, and text labels “Predicted Progressor” and “Predicted Control” indicate logistic regression binary predictions either side of the classification boundary. **(A–E)** Scatter plots for the top pair from each pathway. **(F)** ROCs for each pair logistic regression model classifying all GC6-74 samples. AUCs for each model are shown in the legend, with 95% CIs in parentheses.

To complement the data-driven transcriptional/metabolic discovery analysis, we determined whether a canonical pathway knowledge-driven integration of transcriptional and metabolomic profiles would reveal additional immunometabolic TB risk signatures. Metabolites (*n* = 195) and transcripts (*n* = 5,892) previously selected for global (t-m) complementarity analysis (Table 2) were separately tested for over-representation in canonical metabolic pathways defined in the Kyoto Encyclopedia of Genes and Genomes (KEGG), (33), and joint (t-m) enrichment *p*-values were then calculated using Fisher's method (34). The analysis identified 5 significantly jointly enriched pathways (Table 3, Table S4) that were driven by a total of 142 TB-progression associated transcripts and 61 TB progression-associated metabolites (Table S5). The most strongly jointly enriched pathway was *Lysosome* (Figure S5A), driven by significant transcriptional up-regulation of lysosomal hydrolases and membrane proteins and the metabolite mannose in TB progressors. In particular, a transcript/metabolite pair combining the lysosomal membrane transporter *NPC2* with mannose was a strong predictor of TB progression [ROC AUC: 0.72 (0.66–0.78), Figures 3A,F, Figure S5A]. The *Aminoacyl-tRNA biosynthesis* (AA-tRNA, Figures 3B,F, Figure S5B) and *protein digestion and absorption* (Figures 3C,F, Figure S5C) pathways were also strongly jointly enriched, driven by significant TB progression-associated down-regulation of multiple free serum amino-acids. In particular, the combination of arginine with *WARS* (tryptophanyl-tRNA synthetase) was the strongest complementary TB-associated pairing in the AA-tRNA pathway [ROC AUC: 0.7 (0.64–0.77); Figures 3B,F], and the combination of alanine with *SLC9A3* [ROC AUC: 0.71 (0.65–0.77); Figures 3C,F] was the most predictive (t-m) pair for the protein digestion pathway. The *glutathione* pathway (Figure 3D, Figure S5D), relating to the production of the antioxidant glutathione and other gamma-glutamyl amino-acids, and the *sphingolipid* pathway (Figure 3E, Figure S5E) were also implicated by the joint metabolomics (t-m) enrichment analysis. The combination of the gene *LAP3* with the glutamate precursor 5-oxoproline formed the optimal predictive *glutathione* pathway pair [ROC AUC: 0.72 (0.65–0.78), Figures 3D,F]. In the *sphingolipid* pathway, the combination of the ceramidase *ACER3* and the sphinganine precursor amino-acid serine leads to the strongest pair-predictor of progression [ROC AUC: 0.68 (0.62–0.65), Figures 3E,F].

**Table 3:** KEGG pathways that significantly enriched for TB progression-associated transcripts and metabolites.

**Name**	**KEGG mapID**	**Significant metabolites in pathway**	**All metabolites in pathway**	**Significant transcripts in pathway**	**All transcripts in pathway**	**Combined *P*-value**
Lysosome	map04142	1	1	61	116	0.00
Aminoacyl-tRNA biosynthesis	map00970	8	21	18	41	0.03
Protein digestion and absorption	map04974	8	26	24	48	0.03
Sphingolipid metabolism	map00600	2	5	20	35	0.03
Glutathione metabolism	map00480	4	8	19	41	0.04

### Integration of Pathway-Based Signatures Leads to Improved Prediction of Progression to Active TB in HHCs

**Figure 4:**
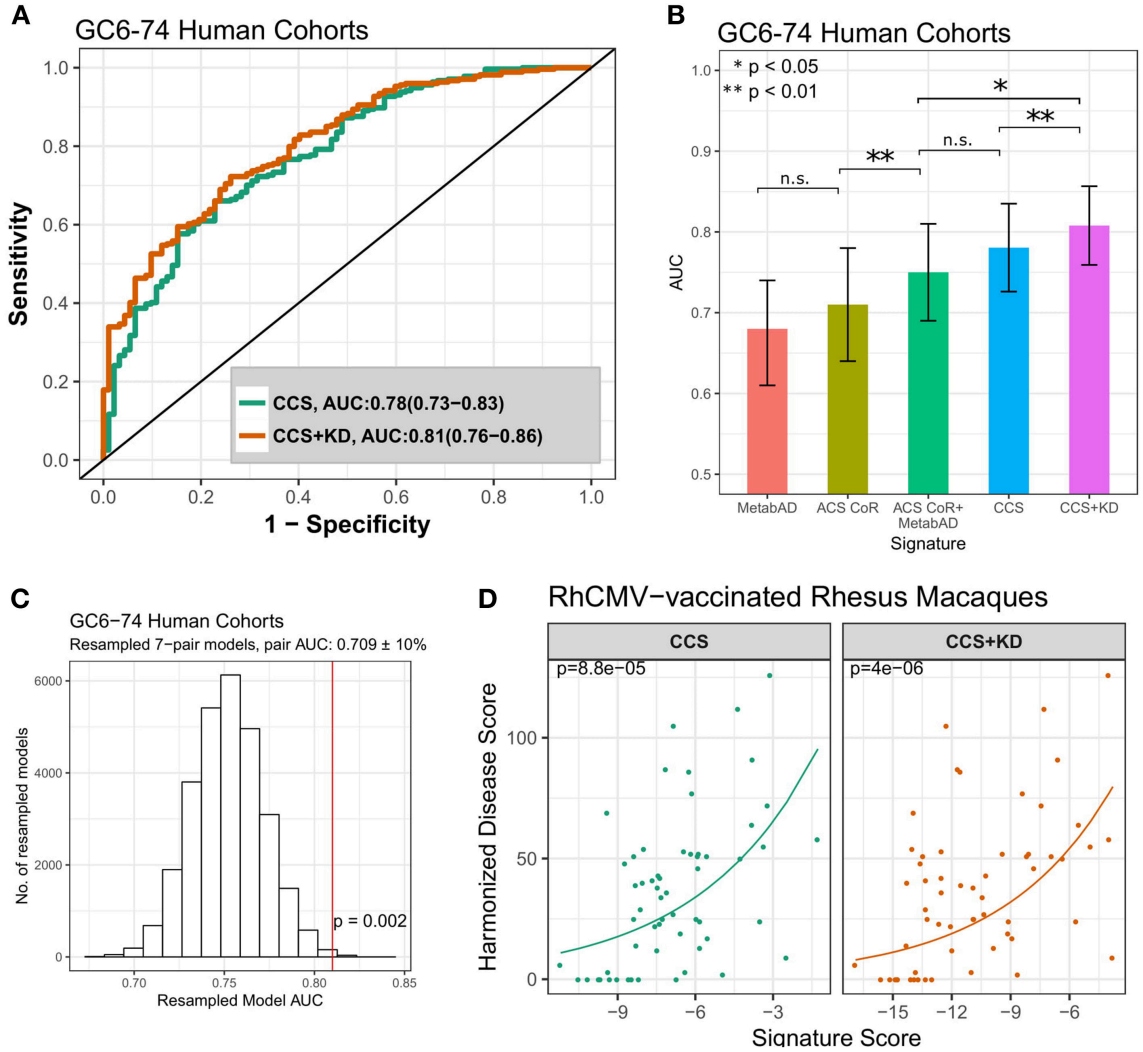
Comparison of the pathway-derived signatures to previously discovered signature of risk of TB progression. **(A)** ROC curves for the pathway-derived CCS and CCS+KD signatures on all GC6-74 samples. **(B)** Comparison of the ROC AUCs of the external signatures (MetabAD and ACS CoR), the combined ACS CoR + MetabAD, and the pathway-based signatures (CCS, CCS+KD). Error bars represent 95% confidence intervals around the AUC. **(C)** Distribution of model AUCs from randomly resampled transcript-metabolite pairs with similar AUCs to pairs in the CCS+KD model. AUC of the CCS+KD signature is indicated by vertical red line. *P*-value indicates the proportion of resampled models with AUC > CCS+KD. **(D)** Scatter plots of CCS and CCS+KD signature scores vs. harmonized disease score in two RhCMV-vaccinated rhesus macaque studies after *M.tb* challenge. Poisson regression was used to determine the relationship between signature score, measured 28 days post-challenge and harmonized disease score at time of necropsy. Solid lines represent Poisson regression fits to the harmonized disease score for CCS and CCS+KD, respectively. *P*-values shown in the top left of each plot indicate significance of association between signature score and harmonized disease score.

We next sought to determine whether the novel knowledge-driven metabolomics (t-m) signatures generated by the joint metabolic pathway enrichment analysis could be combined to yield a more accurate predictor for TB progression. This analysis is driven by our hypothesis that improved knowledge of the biological processes underlying TB progression would result in better prediction—an argument that has been made for omics-based signatures for vaccine immunogenicity and efficacy [34]. We constructed a Composite Canonical metabolic pathway enrichment-based Signature (CCS) that was composed of the sum of scores from the optimal (t-m) pairs derived from each of the five jointly enriched canonical metabolic pathways (described above; Table 4, Figure 3). The ROC of the CCS signature for predicting TB progression in the GC6-74 HHC cohort is shown in Figure 4A, and surpasses the AUC obtained for the ACS CoR, MetabAD, or the combination of the two. We further evaluated whether the CCS signature could be enhanced by the knowledge-driven (KD) metabolomics (t-m) signatures that were identified by the comprehensive correlation analysis—i.e., the SLC25A20-eicosenoate and SOCS1-cortisol pairs (Figure 2C). We termed this signature the CCS+KD signature. Combining the KD with the data-driven multi-omics signatures resulted in further improved discrimination of TB progressors from controls in GC6-74 (CCS+KD ROC AUC=0.81; Figures 4A,B). Although direct comparison of the significance of the CCS+KD and the ACS-CoR signature may be biased by the fact that the CCS+KD signature does not represent a blind prediction on the GC6-74 dataset, overfitting of CCS+KD is limited by its small size (7 genes, 7 metabolites), and use only of validated biological pathways to construct the signature. Nevertheless, to test whether the improvements in predicting TB progression obtained with the CCS+KD signature was significant, we performed a bootstrapped resampling comparison of the signatures. This showed that the CCS+KD signature significantly outperformed the previously published ACS-CoR signature on all samples (*p* = 0.02, Figure 4B). Improved performance was also observed on samples taken within 6 months of TB progression, and on each study site (South Africa, The Gambia, Ethiopia) taken individually (Figures S2, S6).

**Table 4:** Transcript-metabolite pairs most strongly associated with TB progression from each significantly enriched KEGG pathway.

	**Metabolite**	**Gene**	**Model fit AUC**	**AUC lower 95% CI**	**AUC upper 95% CI**
Lysosome	Mannose	NPC2	0.72	0.66	0.78
Aminoacyl-tRNA biosynthesis	Arginine	WARS	0.7	0.64	0.77
Protein digestion and absorption	Alanine	SLC9A3	0.71	0.65	0.77
Sphingolipid metabolism	Serine	ACER3	0.68	0.62	0.75
Glutathione metabolism	5-oxoproline	LAP3	0.70	0.65	0.78

Because a further independent human test set was not available to validate the CCS+KD signature, we compared the signature to predictors based on randomly-selected sets of complementary (t-m) pairs to determine whether the specific combination of gene signatures from distinct metabolic pathways resulted in increased accuracy compared to randomly selected predictive (t-m) pairs. These random pairs were selected so that each individual random pair had similar predictive ability to the pairs making up the composite pathway based predictor. The 7-pair combined pathway model was in the top 0.2% of results (*p* = 0.002) (Figure 4C), indicating that using canonical pathway knowledge to guide signature design yields improved predictive performance.

Finally, we assessed the association between the CCS and CCS+KD signature scores at 28 days post challenge and the RhCMV/TB trial harmonized disease outcome scores (as described above). Strong associtions were observed, with performance being similar for CCS and CCS+KD signatures (CCS: *p* = 8.8 × 10^−5^, τ = 0.356; CCS+KD: *p* = 4.0 × 10^−6^, τ = 0.359 Figure 4D). Both CCS and CCS+KD showed a stronger correlation than was observed for either of the single-omic ACS-CoR or MetabAD signatures (Figure 1C), however only CCS+KD showed a lower model *p*-value for the association. This was despite the non-detection of the SLC9A3 gene in the RM transcriptional data, which required the omission of the alanine/SLC9A3 pair, representing the protein digestion and absorption pathway (Table 4), from the both CCS and CCS+KD scores. Signature performance was driven by the highly significant associations of the arginine/WARS (*p* = 4.3 × 10^−7^, τ = 0.43), cortisol/SOCS1 (*p* = 7.4 × 10^−6^, τ = 0.28) and 5-oxoproline/LAP3 (5.8 × 10^−5^, τ = 0.37) pairs (Figure S8). This approach demonstrates that the biologically interpretable pathway-driven multi-omic signature, based on simple (t-m) pairs, outperforms the existing single-omic signatures for prospectively capturing the spectrum of TB-associated disease that will be observed in M.tb-challenged RM that experience varying degrees of protection mediated by the vaccine RhCMV/MTB.

## Discussion and Conclusions

Improved biosignatures to identify the HHCs of TB cases likely to progress to active disease are urgently needed. Such biosignatures can serve to prioritize at-risk individuals for closer monitoring and targeted prophylactic treatment and to identify high-risk individuals suitable for enrollment in TB vaccine and drug trials (35). Combined transcriptional and metabolomic analyses of blood samples from HHC cohorts have the potential to reveal these predictive biomarkers and simultaneously identify immunometabolic inflammatory processes associated with TB progression. This could also enable development of novel host-directed therapies for TB (36, 37). In the current study, we performed transcriptional and metabolomic analyses of HHCs from multiple African sites in the GC6-74 cohort and demonstrated that these two platforms provide complementary information for predicting TB disease progression. We further integrated these platforms with validated immunometabolic pathway information to generate accurate and functionally interpretable signatures of TB progression. We also demonstrate that while existing signatures of TB progression prospectively correlate strongly with post-necropsy measures of TB-induced lung pathology and bacterial growth measured in the RhCMV/TB vaccine challenge studies, multi-omic signatures show further improved performance. The validation of these multi-omic signatures in both humans and rhesus macaques is noteworthy, as it reveals a universal set of *M.tb* progression pathways in human and RM, underscoring the utility of the RM model to explore the early response to TB in humans.

In our first analysis, we observed that combining a pre-existing transcriptional signature of risk for TB progression [ACS-CoR (28)] with a diagnostic metabolomic signature derived from an independent cohort [MetabAD (25)] yielded a significant improvement in predicting TB progression and showed improved correlation with disease pathology. While our previously-reported ACS CoR demonstrated a sensitivity of 42% at a specificity of 80% for identifying individuals who progress to active disease up to 2 years after the initial HHC exposure, the combined signature (ACS CoR + MetabAD) achieved 56% sensitivity while maintaining 80% specificity on the same samples. This improvement in prediction accuracy allows detection of the majority of TB progressors while maintaining comparatively high specificity. This is important, given that the vast majority of TB exposed individuals do not progress to active disease. As such, the combined ACS CoR + MetabAD signature may serve for therapies in which a rule-in test is appropriate. Importantly, the Stop TB Partnership target product profile for a progression signature (38) is achieved by the ACS CoR (39, 40) for identifying individuals who will progress to active TB in the following 12 months. The ability to improve ACS CoR by combining it with MetabAD will expand the potential clinical relevance of this signature.

By taking data-driven (correlation) and knowledge-driven (pathway) approaches to integrate the GC6-74 transcriptomics and metabolomics data in the context of TB progression, we identified novel functionally-interpretable signatures that demonstrated the potential to predict TB with higher accuracy. While previously reported blood-based signatures of TB disease and TB risk (14–23, 25, 26, 28) largely implicate interferon-driven changes in transcriptional activity (41), the data-driven CCS and CCS+KD signatures identified a broader range of biological functions. This increase in pathway diversity may derive, in part, from the potential of plasma and serum metabolomic profiling to quantify metabolites that are released into bloodstream from tissues throughout the body, not solely limited to blood cells (which is the case for whole blood based transcriptomics), and potentially including the lung and TB granuloma themselves. We identify several key immunometabolic pathways associated with TB disease progression in GC6-74 HHCs. Among the pathways implicated by the CCS signature is the fatty-acid metabolism network. This pathway is of critical importance in TB progression as *M.tb* favors fatty-acids as its cellular nutrient source (42), and *M.tb* itself has roughly 250 genes dedicated to fatty acid metabolism, a higher proportion than any other micro-organism (43).

The CCS+KD signature also implicates key immune regulatory pathways in TB progression, particularly strong correlations between levels of the metabolite cortisol and the genes *SOCS1* and *DDIT4*, all three of which are upregulated in TB progressors. Cortisol is a glucocorticoid steroid hormone that induces apoptosis through activation of the glucocorticoid receptor, and it has been shown that inhibition of *mTOR* with rapamycin sensitizes lymphoid cells to glucocorticoid receptor mediated apoptosis (44). *SOCS1* (Suppressor of Cytokine Signaling 1) is a negative regulator of signaling in the JAK/STAT pathway, which acts downstream of interferon-gamma. *SOCS1*, in its role as a repressor of the JAK/STAT pathway, shows extensive cross-talk with *mTOR* in response to interferon signaling (45). *DDIT4* (DNA-damage inducible transcript 4), regulates p53/TP53-mediated apoptosis via the mTORC1 complex in response to DNA damage and hypoxic stress (46). The optimal SOCS1-Cortisol (t-m) pair in this subnetwork is the strongest individual predictor in the combined signature, underscoring the key role of the *mTOR* and JAK/STAT immune signaling pathways in TB progression. The *lysosome* pathway was among the five pathways implicated by the knowledge-driven analysis. This may reflect the established ability of *M.tb* to prevent formation of the phagolysosome complex (47, 48) for protection from degradation by lysosomal enzymes and autophagy, thus decreasing antigen presentation (49), and facilitating subsequent *M.tb* escape to the cytosol (50). The analysis similarly implicated the *protein digestion* pathway, which was strongly down-regulated during TB progression, particularly *SLC9A3* Na+/H+ membrane transporter protein. Importantly, lysosomal activity depends on the maintenance of an acidic milieu in the phagolysosome, and the main role of *SLC9A3* is to regulate intracellular pH by transporting H+ out of the phagolysosome. *M. tb* also plays an active role in counteracting the phagolysosomal maturation and subsequent acidification (51). These were accompanied by the related *amino-acyl tRNA* pathway, which reflects the significant decrease of amino-acid levels in progressors vs. controls. Free amino-acids are produced by the degradation of proteins in the lysosome. Recently, it has been observed that inhibition of *mTOR* strongly reduces the efflux of amino-acids from the lysosome (52). Thus, mechanistic linkages exist between four of the pathways implicated here in TB progression (*lysosome, protein digestion, amino-acid tRNA*, and *cortisol signaling*).

The *glutathione* pathway, relating to the production of the antioxidant glutathione and other gamma-glutamyl amino-acids, was also selected as part of the CCS signature. Consistent with the observed upregulation of protein-degradation enzymes in the lysosome, leucine amino peptidase 3 (*LAP3*) was strongly upregulated in TB progressors, and this enzyme formed part of the optimal predictive (t-m) pair in this pathway. Also notable was the observed down-regulation of glutathione peroxidase 2 (*GPX2*) in progressors. *GPX2* helps protect cells against oxidative stress by catalyzing glutathione-mediated reduction of peroxides. While these oxidative peroxides can damage the cell, release of reactive oxygen species by macrophages plays a critical role in bacterial killing (53). Reduction in *GPX2* levels is consistent with allowing a higher degree of bacterial killing accompanied by increased host cell damage. Counteracting this process by supplementation with N-acetylcysteine to increase glutathione levels during TB treatment has recently been shown to be associated with faster sputum negativity and reduced lung cavity size (54). This example indicates the potential of this pathway-driven analysis to reveal targets for host directed therapies. Our analysis also implicated the *Sphingolipid metabolism* pathway, an established mediator of the host response to TB (55–57). Sphingolipids are key building blocks of cell membranes which also play important roles in immune signaling and represent major constituents of the mucus secreted by lung alveolar epithelial cells (57). In particular, sphingosine-1-phosphate is involved in the induction of antibacterial activity in macrophages that participate in the control of *M.tb* (55). Inhibition of translocation of cytosolic *SK1* to the phagosome membrane is associated with survival of *M.tb* (56). Furthermore, *M.tb* may manipulate host sphingolipid metabolism to enhance its persistence and replication (57).

The approach described in this work can, in principle, be applied to other, similar diseases. Leprosy, another mycobacterial disease, also often remains asymptomatic in the host for years after the initial infection. Thus, a signature of risk post-exposure could be of clinical value. Gene-expression studies have been performed on several human leprosy disease cohorts, looking at samples taken from patients; including leprosy skin lesions (58), Schwann cells from nerve biopsies (59), and from PBMCs (60). A key limitation of these studies is that samples are from individuals who have already succumbed to active disease. The development of a prospective signature of leprosy risk would require the recruitment and follow-up of a high-risk population in order to search for biomarkers apparent before disease onset. Lessons learned from TB risk cohorts in this study suggest immune processes associated with leprosy may also be evident in blood prior to the onset of symptoms, and transcriptional studies of active disease may be of use to guide the discovery of a potential leprosy risk signature.

The integrated transcriptomic and metabolomic approaches applied here have allowed characterization of biological processes that are coordinated simultaneously inside and outside the cell, which cannot be captured by transcriptional profiling alone. Intriguingly, the set of processes identified here as significantly involved in progression in certain aspects mirrors events occurring in *M. tb* itself. Galagan et al. (61) have previously revealed a direct link between the hypoxic stress response, fatty acid catabolism, lipid biosynthesis, and protein degradation in the *M.tb* transcriptional regulatory network.

An unanswered question still exists: what is the precise protective or pathogenic role associated with each of these pathways? Transcriptomic and proteomic investigation of TB-infected adolescents suggests that there exists a sequential modulation of immunological processes leading to TB progression (41), consistent with TB progression from incipient, to subclinical, to active disease. However, it remains unclear whether our power to predict TB progression is derived from observing a normally effective immune response to TB be overcome by high bacterial load, or whether differential regulation of the pathways identified here represents a failure of particular immune mechanisms to respond adequately to *M.tb*. Recent studies have noted a continuum of granulomas at different developmental stages including solid granulomas, necrotic granulomas and caseous granulomas, and the decisive impact of the range of granuloma microenvironments in a single patient potentially showing a range of outcomes from sterilizing immunity to loss of control (62, 63). This suggests that analysis of biological samples taken close to or from the granuloma itself may be required to better understand the precise protective or pathogenic role of diverse host responses to TB. In addition, a greater knowledge of pathways associated with progression can lead to novel host-directed approaches to improve treatment outcomes, and suggest biological mechanisms underlying granuloma-specific loss-of-control. Future analysis may expand this strategy by integrating other complementary high throughput assessments of host inflammatory and metabolic processes, including cellular and serum proteomics, intra-cellular metabolomics, and focused single-cell analysis of macrophages and T-cells (64).

## Materials and Methods

The study design, sample acquisition, RNAseq and metabolomic profiling techniques applied here have been described in detail elsewhere (25, 29, 30, 40). These methods are summarized below.

### Household Contact Study Design and Sample Acquisition

This study includes cohorts from four geographic sites, all with a prospective longitudinal design to identify prospective correlates of risk of TB disease. The household contact study design included participants from four African sites: South Africa, The Gambia, Ethiopia, and Uganda, as part of the Bill and Melinda Gates Grand Challenges 6–74 study. The GC6-74 parent cohort consisted of 4,466 HIV-negative participants, aged 10–60 years, with no clinical signs of active TB disease. Participants were HHCs of a TB index case, who was at least 15 years old, with a confirmed positive sputum smear for acid fast bacilli. HHCs were enrolled within no more than 2 months of the index case being diagnosed with active TB.

HHCs who progressed to active TB disease between 3 and 24 months from recruitment were considered progressors. Active TB in progressors was diagnosed by microbiological confirmation of *M.tb* in sputum samples in all except 7 individuals, who were diagnosed based on clinical symptoms, chest and other radiographs (CXR) consistent with TB and response to chemotherapy, without microbiological confirmation (29). HHCs diagnosed with active TB disease within 3 months of enrolment were excluded from further analysis. Each progressor was matched to 4 controls who remained healthy during follow-up. Matching was done by site, age category, sex, and year of recruitment category. Age categories included 4 categories: <18, 18–25, 25–36, and >36 years of age, and year of enrolment had 3 categories: 2006/2007, 2008, and 2009/2010. For all sites, samples were collected at enrolment (baseline), and at 6 and 18 months post-enrolment, provided the participant remained free of active TB at the time of sampling.

### RhCMV/TB RM Study Design and Sample Acquisition

RM disease outcome data was obtained from the previously published RhCMV/TB vaccine trial (32). This trial comprised RMs from two independent challenge studies, including those vaccinated with RhCMV/TB candidate vaccines, BCG and unvaccinated RMs. RM counts for each experimental group were 23 from Study 1, and 36 from Study 2. Thirty three RMs were vaccinated with a RhCMV/TB candidate vaccine; 7 were vaccinated with both RhCMV/TB and BCG; 13 were unvaccinated, and 6 vaccinated with BCG only.

### RNAseq Profiling

RNAseq was carried out as previously described (28, 32). PAXgene blood RNA samples (Beckton Dickinson, New Jersey, USA) from Uganda and Ethiopia and extracted RNA from the Gambia were shipped for processing at the University of Cape Town. RNA was extracted from blood using the PAXgene Blood RNA kit (Qiagen, Germantown, MD, USA), and separated into aliquots for local quality control, RNA-sequencing and qRT-PCR. Quantification of RNA and initial quality control were performed using the NanoDrop 2000™ spectrophotometer (ThermoFisher Scientific, Waltham, MA, USA) to measure concentrations and 260/280 ratios, followed by sampling on the Agilent 2100 Bioanalyzer (Agilent, Santa Clara, CA, USA) to determine RNA Integrity Number (65). TruSeq cDNA library preparation (Illumina, San Diego, CA, USA) from a minimum of 200 ng RNA samples of RIN ≥ 7 was sequenced at the Beijing Genomics Institute (BGI, Shenzhen, China), at 60 million 50 bp paired-end reads using Illumina HiSeq-4000 sequencers. Gsnap (66) software was used to align read pairs to the hg19 human genome. Further analysis was done using the gene count table, normalized with edgeR. Transcriptional profiles are available on NCBI GEO for GC6-74 samples (GSE94438), and RhCMV/TB RMs (GSE102440).

### Metabolomic Profiling

Metabolomic profiling was performed by Metabolon, Inc. as described previously (67), using either participant plasma (Ethiopia, The Gambia) or serum (Uganda, South Africa) samples. For RMs, plasma was collected as previously described (32). Plasma samples from Study 1 and Study 2 were analyzed in two separate batches ~6 months apart. Prior to metabolomics analysis, RM plasma samples were rendered non-infectious by sterile filtering twice using sterile 25 mm Pall acrodisc PF syringe filters with stupor membrane (prod. #4187).

Plasma and serum samples were analyzed in concert with a pool of normalization control plasma extensively characterized by Metabolon. Samples were analyzed using three mass-spectrometry pipelines: ultra high performance liquid chromatography-tandem mass spectrometry (UPLC-MS/MS; positive mode), UPLC-MS/MS (negative mode), and gas chromatography–mass spectrometry (GC-MS). The UPLC-MS/MS pipeline used a Waters ACQUITY UPLC (BEH C18-2.1 x 100 mm, 1.7 μm) and a Thermo Scientific Q-Exactive high resolution/accurate mass spectrometer interfaced with a heated electrospray ionization (HESI-II) source and Orbitrap mass analyzer. The GC/MS pipeline used a Thermo-Finnigan Trace DSQ GC/MS with electron impact (EI) ionization.

Metabolites were identified by automated comparison of the ion features in the experimental samples to a reference library of >4,000 chemical standard entries that included retention time, molecular weight (m/z), preferred adducts, and in-source fragments as well as associated MS spectra and curated by visual inspection for quality control using software developed at Metabolon (68). Additional mass spectral entries have been created for structurally unnamed biochemicals (>5,000 in the Metabolon library), which have been identified by virtue of their recurrent nature (both chromatographic and mass spectral). These compounds have the potential to be identified by future acquisition of a matching purified standard or by classical structural analysis.

Peaks were quantified using area-under-the-curve. Raw area counts for each metabolite in each sample were normalized to correct for variation resulting from instrument inter-day tuning differences by the median value for each run-day, therefore, setting the medians to 1.0 for each run. Missing values were imputed with the observed minimum after normalization.

Metabolomic profiles for GC6-74 samples are available from Metabolomic Workbench (69) (www.metabolomicsworkbench.org, ID: PR000666). Metabolomic profiles for RhCMV/TB samples are attached as Table S6.

### Development of Metabolomics Disease Signature (MetabAD)

The metabolomics active disease cohort obtained by Weiner et al. (25) was used to train and parameterize the metabolomics active disease model. These samples are available as part of the R tmod (70) package as the *tbmprof* dataset. Metabolites detected in GC6-74 plasma and serum samples that also had values available in the active disease dataset were considered for model inclusion (207 metabolites). A random forest model was trained using the R (71) caret (72) package, with performance assessed by leave-one-out cross validation. Metabolite model importance to the model was ranked by contribution to prediction error using the caret varImp function, and the model retrained on the top 100 features only. This was repeated recursively to shrink the model to 50 and 25 metabolites. This 25-metabolite signature was used for all further analyses.

### Receiver-Operating Characteristic (ROC) Analysis

The R package pROC (73) (function: roc) was used to calculate ROC curves by applying a set of thresholds to numeric predictions from predictive models to predict the progressor/non-progressor status of the samples, and then calculating the sensitivity and specificity of the predictor at each threshold. 95% confidence intervals (CI) around the AUC were estimated using 2000 bootstrapped replicates as implemented in pROC, and ROC curves with a lower 95% confidence interval above 0.5 were considered statistically significant. ROC curves were plotted using the R ggplot2 (74) package. The optimal classification threshold for binary classification was chosen from the ROC curve by identifying the point on the ROC curve with the smallest Euclidian distance to perfect classification (sensitivity = 1 and specificity = 1).

Significance of combining the ACS CoR and MetabAD signatures was assessed by fitting a logistic regression model to ACS CoR alone, MetabAD alone, and ACS CoR + MetabAD and using the R anova.glm(test = “Chisq”) function to test for a significant reduction in residual deviance for the Combined model vs. both ACS CoR and MetabAD alone.

### Poisson Modeling of RhCMV Study Data

Transcriptomic, metabolomics, and harmonized disease outcome data data from the RhCMV/TB trial were used to develop regression models of association between harmonized disease score “Outcome” and (t-m) signature scores “Signature_Score.” As disease outcome scores were strictly non-negative and derived from lung pathology and TB culture count scores, Poisson regression was chosen as the appropriate modeling approach. Two forms of models were fit to evaluate goodness of fit to the data, and a “Study” term was included in the modeling in order to exclude technical effects related to the separately performed transcriptional or metabolomic profiling of the particular vaccine study (Study 1 or Study 2).

Model 1: Outcome ~ Study

Model 2: Outcome ~ Study + Signature_Score

Wald tests [R function: waldtest; library: lmtest (75)] using the regression coefficient covariance matrix provided by the heteroscedasticity-consistent covariance estimation (R library: sandwich) were performed comparing the two (above) models generated for each signature. Thus, the resulting *P*-value measures the study-invariant *P*-value of the association of Outcome and Signature_Score. The correlation between study adjusted Signature_Score and Outcome was calculated as Kendall's tau (τ).

All plots showing the results of modeling the relationship between Signature_Score and Outcome have been adjusted to remove the effect of the Study coefficient.

### Logistic Regression Modeling

Logistic regression models of the form progression ~ (gene|metabolite) were fit for each individual metabolite and gene, using the R glm() function. χ^2^ *p*-values were calculated using the R anova.glm() function. To assess complementarity with ACS COR, logistic regression models of the form (1) progression ~ ACS CoR and (2) progression ~ ACS CoR + metabolite were fit. Significant complementation was defined as model (2) showing significantly better fit to the data (*p* < 0.05) than model (1) calculated using anova.glm(test = “Chisq”). For each gene-metabolite pair, a model of the form (1) progression ~ gene + metabolite was compared to both (2) progression ~ gene and (3) progression ~ metabolite. Complementarity was defined as model (1) being significantly better than both models (2) and (3) as measured by anova.glm().

### Transcript-Metabolite Correlations

Transcript-metabolite correlations were calculated for matched samples using Spearman's rho. *P*-values for these correlations were calculated using Fisher's transformation, and a false-discovery rate correction applied. Significant overlap of correlated pairs of genes and metabolites significant at FDR < 0.01 were compared between GC6-74 HHC and KORA F4 using the hypergeometric test.

### Joint Pathway Analysis

KEGG IDs for each transcript were obtained using the biomaRt (76) and KEGGREST (77) R packages. KEGG pathway annotations for each transcript were obtained using the Bioconductor org.Hs.eg.db (78) package. KEGGREST was used to obtain KEGG pathway IDs for each metabolite. The number of significant genes and significant metabolites from the GC6-74 HHC datasets mapped to each pathway was determined, and compared with the total number of genes and metabolites mapped. The hypergeometric test was used separately to determine pathways enriched in significant (1) genes and (2) metabolites. These two *p*-values were combined using Fisher's method to determine an overall *p*-value for the pathway.

### Joint Metabolic Pathway Enrichment-Based Logistic Regression Models

Logistic regression models were fit, as above, for each gene/metabolite pair in each significant pathway, with the single most predictive pair from each pathway being selected. Predictions from each selected gene/metabolite pair model were summed to form the final pathway predictor.

### Significance Testing of Pathway-Enrichment-Based Model

The pathway based model was created by selecting the most predictive and synergistic (as described above) (t-m)-pair for each significant KEGG pathway. The top synergistic pairs from each correlation subnetwork (if a synergistic pair was identified) were also included. Predictive performance of the individual pairs in the signature was referred to as “*pair-ROC*.” The overall ROC for the signature was calculated by summing the logistic-regression predictions for each individual pair to construct an overall ROC curve (“*sum-ROC*”).

Significance of this model was calculated by repeatedly randomly sampling the same number (N) of synergistic pairs as were included in the model from the global set of synergistic pairs. Here *N* = 7 = the total number of pairs in the composite pathway predictor. In order to ensure comparable performance of randomly sampled pairsets to those in the pathway model, the mean *pair-ROC* for each pairset was required to be within ± 10% of the mean *pair-ROC* for the pathway model (mean composite AUC = 0.709). The combined AUC for the random predictor was then calculated by summing the individual pairs, in the same way as for the pathway predictor (see Figure S7). Significance was calculated as *p* = 1—(fraction of randomly sampled pair models with lower *sum-ROC*).

Significance comparison of the combined pathway based model to the ACS CoR model was done using a bootstrapped comparison implemented in the pROC roc.test() function.

## Data Availability

All datasets generated for this study are included in the manuscript with the exception of the rhesus macaque metabolics data which is included as Table S6.

## Ethics Statement

All study sites adhered to the Declaration of Helsinki and Good Clinical Practice guidelines. Ethical approvals were obtained from institutional review boards (IRBs) and all study participants provided written informed consent. For all sites, adult participants, or legal guardians of participants aged 10–17 years old, provided written or thumb-printed informed consent to participate after careful explanation of study aims and any potential risks. The relevant IRBs and ethical approvals were: for the South African study site, the Stellenbosch University Institutional Review Board (N05/11/187); for the Ugandan study site, the Uganda National Council for Science and Technology (MV 715) and University Hospitals Case Medical Center (12-95-08); for the Ethiopian site, the Armauer Hansen Research Institute (AHRI)/All Africa Leprosy, TB and Rehabilitation Training Center (ALERT) (P015/10); and for the Gambian study site, the Joint Medical Research Council and Gambian Government (SCC.1141vs2). Thumb-printed indication of informed consent was explicitly included as acceptable in the IRB-approved Informed Consent Form (ICF) for the Gambian site. None of the Ugandan study participants used thumb-printed confirmation of informed consent. For the South African and Ethiopian sites, we obtained letters from the relevant IRBs explicitly approving the use of thumb-printed informed consent.

## Author Contributions

FD, JW, SS, ET, JM, SmS, TS, SK, and DZ: study approach and data interpretation; JW, SH, DT, SaS, JM, GT, SP, DD, MA, JS, HD, TS, LP, GW, SK, and DZ data acquisition; JS, DZ, GW, SK, TS, HD, LP, and TO: project conception. All authors contributed to drafting, writing and revising the manuscript, and approved the final submission.

### Conflict of Interest Statement

The authors declare that the research was conducted in the absence of any commercial or financial relationships that could be construed as a potential conflict of interest.

## Supplementary Material

Figure S1 Overview of the multi-step analytical strategy employed to test whether integration of blood transcriptional profiling with serum metabolomic profiling can provide new understanding of disease processes and enable improved prediction of TB progression. ACS CoR, Adolescent Cohort Signature Correlate of Risk; MetabAD, Metabolomics Active Disease signature; CCS, Composite Canonical Signature; CCS+KD, Composite Canonical Signature plus Knowledge Driven pathways.

Figure S2 ROC curves of ACS-CoR, MetabAD, and combined ACS CoR + MetabAD signature predictions on subsets on the GC6-74 samples. **(A)** Progressors within 6 months of active TB vs. healthy controls. **(B–D)** predictions of specific samples from the South African, Gambian, and Ethiopian sites, respectively. **(E)** *P*-values (single-tailed Delong test) indicating whether the improvement in prediction for the ACS CoR+MetabAD model over either individual model is significant for each subset.

Figure S3 Network plot of all transcript/metabolite pairs previously identified as correlated in KORA F4 that are also significantly correlated in GC6-74 samples. Transcript nodes are shown as diamonds, metabolite nodes as circles, with significant correlations indicated by edges linking transcripts and metabolites. Positive correlations between metabolites and transcripts are shown as green edges and negative correlations as red. Darker shades indicate stronger correlations (legend shown bottom left). Transcripts and metabolites that showed significant association with TB progression were shaded in purple, with unassociated nodes shaded gray. Darker shades indicate more significant association, according to legend in bottom left.

Figure S4 Concordance of correlations between the KORA F4 and GC6-74 transcript/metabolite pairs. Each point represents a single (t-m) pair significantly correlated in the KORA F4 cohort. The x-axis shows Spearman correlation coefficients for (t-m) pairs from the KORA F4 cohort, and the y-axis shows the equivalent correlations in GC6-74. The red line represents x=y, perfect concordance, and the blue line is the linear best fit.

Figure S5 KEGG pathway maps for each of the significant KEGG pathways shown in Table 3. **(A–E)** Maps for lysosome, aminoacyl-tRNA synthesis, protein digestion and absorption, glutathione metabolism, and sphigolipid metabolism respectively. Significantly up- and down-regulated transcripts are highlighted in red and green, respectively, and significantly up- and down-regulated metabolites are shown in blue and yellow, respectively.

Figure S6 ROC curves for the CCS+KD classifier on subsets of the GC6-74 cohort. Signature performance is shown for progressors within 6 months of active disease, and for each individual study site.

Figure S7 Flow diagram illustrating the resampling procedure used to compare performance of the pathway-derived signature to randomly constructed signatures containing the same number of (t-m) pairs with similar predictive performance.

Figure S8 Scatter plots of individual pairs from the CCS+KD classifier compared to harmonized disease scores from two independent RM vaccine trial studies measured 28 days post-challenge with *M.tb*. Solid lines indicate the best-fit Poisson model, and *p*-values shown indicate Poisson *p*-values for the association between individual pair score 28 days post-challenge and harmonized disease score.

Table S1 Metabolites included in the MetabAD TB diagnostic signature.

Table S2 Summarized binary classifications of the ACS-CoR and MetabAD signatures on the shared GC6-74 dataset. Binary classification thresholds were selected as the point on the ROC curve (Figure 1A) closest to 100% sensitivity and specificity.

Table S3 (t-t), (t-m), and (m-m) pairs that showed significant complementarity in discriminating progressor and control samples.

Table S4 (t-m) pairs associated with TB progression found in all combined transcriptional/metabolic KEGG pathways.

Table S5 Progression-associated fold changes of each significant transcript and metabolite mapped to the KEGG pathways listed in Table 3.

Table S6 Metabolite levels in the RhCMV/TB RM study 28 days post *M.tb* challenge.

## References

[1] World Health Organization Global Tuberculosis Report. Geneva (2018). Available online at: http://www.who.int/tb/publications/global_report/en/

[2] Mandalakas AM Starke JR. Current concepts of childhood tuberculosis. Semin Pediatr Infect Dis. (2005) 16:93–104. 10.1053/j.spid.2005.01.001 15825140

[3] Marais BJ Graham SM Cotton MF Beyers N. Diagnostic and management challenges for childhood tuberculosis in the era of HIV. J Infect Dis. (2007) 196(Suppl 1):S76–85. 10.1086/518659 17624829

[4] Pawlowski A Jansson M Skold M Rottenberg ME Kallenius G. Tuberculosis and HIV co-infection. PLoS Pathog. (2012) 8:e,1002464. 10.1371/journal.ppat.1002464 22363214 PMC3280977

[5] Dooley KE Chaisson RE. Tuberculosis and diabetes mellitus: convergence of two epidemics. Lancet Infect Dis. (2009) 9:737–46. 10.1016/S1473-3099(09)70282-8 19926034 PMC2945809

[6] Ronacher K Joosten SA Van Crevel R Dockrell HM Walzl G Ottenhoff TH. Acquired immunodeficiencies and tuberculosis: focus on HIV/AIDS and diabetes mellitus. Immunol Rev. (2015) 264:121–37. 10.1111/imr.12257 25703556

[7] Aibana O Franke MF Huang CC Galea JT Calderon R Zhang Z. Impact of vitamin A and carotenoids on the risk of tuberculosis progression. Clin Infect Dis. (2017) 65:900–9. 10.1093/cid/cix476 28531276 PMC5848231

[8] Verver S Warren RM Munch Z Richardson M Van Der Spuy GD Borgdorff MW. Proportion of tuberculosis transmission that takes place in households in a high-incidence area. Lancet. (2004) 363:212–4. 10.1016/S0140-6736(03)15332-9 14738796

[9] Andrews JR Noubary F Walensky RP Cerda R Losina E Horsburgh CR. Risk of progression to active tuberculosis following reinfection with Mycobacterium tuberculosis. Clin Infect Dis. (2012) 54:784–91. 10.1093/cid/cir951 22267721 PMC3284215

[10] Fox GJ Barry SE Britton WJ Marks GB. Contact investigation for tuberculosis: a systematic review and meta-analysis. Eur Respir J. (2013) 41:140–56. 10.1183/09031936.00070812 22936710 PMC3533588

[11] Jones-Lopez EC Namugga O Mumbowa F Ssebidandi M Mbabazi O Moine S. Cough aerosols of Mycobacterium tuberculosis predict new infection: a household contact study. Am J Respir Crit Care Med. (2013) 187:1007–15. 10.1164/rccm.201208-1422OC 23306539 PMC3707366

[12] Kasaie P Andrews JR Kelton WD Dowdy DW. Timing of tuberculosis transmission and the impact of household contact tracing. An agent-based simulation model. Am J Respir Crit Care Med. (2014) 189:845–52. 10.1164/rccm.201310-1846OC 24559425

[13] Houben RM Dodd PJ. The global burden of latent tuberculosis infection: a re-estimation using mathematical modelling. PLoS Med. (2016) 13:e1002152. 10.1371/journal.pmed.1002152 27780211 PMC5079585

[14] Berry MP Graham CM Mcnab FW Xu Z Bloch SA Oni T. An interferon-inducible neutrophil-driven blood transcriptional signature in human tuberculosis. Nature. (2010) 466:973–7. 10.1038/nature09247 20725040 PMC3492754

[15] Maertzdorf J Ota M Repsilber D Mollenkopf HJ Weiner J Hill PC. Functional correlations of pathogenesis-driven gene expression signatures in tuberculosis. PLoS ONE. (2011) 6:e26938. 10.1371/journal.pone.0026938 22046420 PMC3203931

[16] Maertzdorf J Repsilber D Parida SK Stanley K Roberts T Black G. Human gene expression profiles of susceptibility and resistance in tuberculosis. Genes Immun. (2011) 12:15–22. 10.1038/gene.2010.51 20861863

[17] Maertzdorf J Weiner J III Mollenkopf HJ Network TB Bauer T Prasse A. Common patterns and disease-related signatures in tuberculosis and sarcoidosis. Proc Natl Acad Sci USA. (2012) 109:7853–8. 10.1073/pnas.1121072109 22547807 PMC3356621

[18] Ottenhoff TH Dass RH Yang N Zhang MM Wong HE Sahiratmadja E. Genome-wide expression profiling identifies type 1 interferon response pathways in active tuberculosis. PLoS ONE. (2012) 7:e45839. 10.1371/journal.pone.0045839 23029268 PMC3448682

[19] Bloom CI Graham CM Berry MP Rozakeas F Redford PS Wang Y. Transcriptional blood signatures distinguish pulmonary tuberculosis, pulmonary sarcoidosis, pneumonias and lung cancers. PLoS ONE. (2013) 8:e70630. 10.1371/annotation/7d9ec449-aee0-48fe-8111-0c110850c0c1 23940611 PMC3734176

[20] Kaforou M Wright VJ Oni T French N Anderson ST Bangani N. Detection of tuberculosis in HIV-infected and -uninfected African adults using whole blood RNA expression signatures: a case-control study. PLoS Med. (2013) 10:e1001538. 10.1371/journal.pmed.1001538 24167453 PMC3805485

[21] Verhagen LM Zomer A Maes M Villalba JA Del Nogal B Eleveld M. A predictive signature gene set for discriminating active from latent tuberculosis in Warao Amerindian children. BMC Genomics. (2013) 14:74. 10.1186/1471-2164-14-74 23375113 PMC3600014

[22] Anderson ST Kaforou M Brent AJ Wright VJ Banwell CM Chagaluka G. Diagnosis of childhood tuberculosis and host RNA expression in Africa. N Engl J Med. (2014) 370:1712–23. 10.1056/NEJMoa1303657 24785206 PMC4069985

[23] Tientcheu LD Haks MC Agbla SC Sutherland JS Adetifa IM Donkor S. Host immune responses differ between M. *africanum*- and *M tuberculosis*-infected patients following standard anti-tuberculosis treatment PLoS Negl Trop Dis. (2016) 10:e0004701. 10.1371/journal.pntd.0004701 27192147 PMC4871581

[24] O'neill LA Kishton RJ Rathmell J. A guide to immunometabolism for immunologists. Nat Rev Immunol. (2016) 16:553–65. 10.1038/nri.2016.70 27396447 PMC5001910

[25] Weiner J III Parida SK Maertzdorf J Black GF Repsilber D Telaar A. Biomarkers of inflammation, immunosuppression and stress with active disease are revealed by metabolomic profiling of tuberculosis patients. PLoS ONE. (2012) 7:e40221. 10.1371/journal.pone.0040221 22844400 PMC3402490

[26] Frediani JK Jones DP Tukvadze N Uppal K Sanikidze E Kipiani M. Plasma metabolomics in human pulmonary tuberculosis disease: a pilot study. PLoS ONE. (2014) 9:e108854. 10.1371/journal.pone.0108854 25329995 PMC4198093

[27] Bartel J Krumsiek J Schramm K Adamski J Gieger C Herder C. The human blood metabolome-transcriptome interface. PLoS Genet. (2015) 11:e1005274. 10.1371/journal.pgen.1005274 26086077 PMC4473262

[28] Zak DE Penn-Nicholson A Scriba TJ Thompson E Suliman S Amon LM. A blood RNA signature for tuberculosis disease risk: a prospective cohort study. Lancet. (2016) 387:2312–22. 10.1016/S0140-6736(15)01316-1 27017310 PMC5392204

[29] Suliman S Thompson E Sutherland J Weiner Rd J Ota MOC Shankar S Four-gene Pan-African blood signature predicts progression to tuberculosis. Am J Respir Crit Care Med. (2018) 197:1198–208. 10.1164/rccm.201711-2340OC PMC6019933 29624071

[30] Weiner J III Maertzdorf J Sutherland JS Duffy FJ Thompson E Suliman S. Metabolite changes in blood predict the onset of tuberculosis. Nat Commun. (2018) 9:5208. 10.1038/s41467-018-07635-7 30523338 PMC6283869

[31] Duffy FJ Thompson E Downing K Suliman S Mayanja-Kizza H Boom WH. A serum circulating miRNA signature for short-term risk of progression to active tuberculosis among household contacts. Front Immunol. (2018) 9:661. 10.3389/fimmu.2018.00661 29706954 PMC5908968

[32] Hansen SG Zak DE Xu G Ford JC Marshall EE Malouli D. Prevention of tuberculosis in rhesus macaques by a cytomegalovirus-based vaccine. Nat Med. (2018) 24:130–43. 10.1038/nm.4473 29334373 PMC5909823

[33] Kanehisa M Goto S Sato Y Kawashima M Furumichi M Tanabe M. Data, information, knowledge and principle: back to metabolism in KEGG. Nucleic Acids Res. (2014) 42:D199–205. 10.1093/nar/gkt1076 24214961 PMC3965122

[34] Elston RC On fisher's method of combiningp-values. Biometric J. (1991) 33:339–45. 10.1002/bimj.4710330314

[35] Kaufmann SH Evans TG Hanekom WA. Tuberculosis vaccines: time for a global strategy. Sci Transl Med. (2015) 7:276fs278. 10.1126/scitranslmed.aaa4730 25717094

[36] Zumla A Maeurer M Host-Directed Therapies N Chakaya J Hoelscher M Ntoumi F. Towards host-directed therapies for tuberculosis. Nat Rev Drug Discov. (2015) 14:511–2. 10.1038/nrd4696 26184493

[37] Kaufmann SHE Dorhoi A Hotchkiss RS Bartenschlager R. Host-directed therapies for bacterial and viral infections. Nat Rev Drug Discov. (2017) 17:35–56. 10.1038/nrd.2017.162 28935918 PMC7097079

[38] Seshadri P Denkinger C Draft *Target Product Profile: Test For Progression Of Tuberculosis Infection [Online]*. Foundation for Innovative New Diagnostics (2016). Available online at: https://www.finddx.org/wp-content/uploads/2016/05/TPP-LTBIprogression.pdf (Accessed 17 August 2017).

[39] Kik SV Cobelens F Moore D. Predicting tubserculosis risk. Lancet. (2016) 388:2233. 10.1016/S0140-6736(16)32070-0 27825497

[40] Zak D Scriba TJ Hatherill M Penn-Nicholson A Hanekom W. Predicting tuberculosis risk - Authors' reply. Lancet. (2016) 388:2233–4. 10.1016/S0140-6736(16)31653-1 27825498

[41] Scriba TJ Penn-Nicholson A Shankar S Hraha T Thompson EG Sterling D. Sequential inflammatory processes define human progression from M. tuberculosis infection to tuberculosis disease. PLoS Pathog. (2017) 13:e1006687. 10.1371/journal.ppat.1006687 29145483 PMC5689825

[42] Lee W Vanderven BC Fahey RJ Russell DG. Intracellular Mycobacterium tuberculosis exploits host-derived fatty acids to limit metabolic stress. J Biol Chem. (2013) 288:6788–800. 10.1074/jbc.M112.445056 23306194 PMC3591590

[43] Kinsella RJ Fitzpatrick DA Creevey CJ Mcinerney JO. Fatty acid biosynthesis in Mycobacterium tuberculosis: lateral gene transfer, adaptive evolution, and gene duplication. Proc Natl Acad Sci USA. (2003) 100:10320–5. 10.1073/pnas.1737230100 12917487 PMC193559

[44] Miller AL Garza AS Johnson BH Thompson EB. Pathway interactions between MAPKs, mTOR, PKA, and the glucocorticoid receptor in lymphoid cells. Cancer Cell Int. (2007) 7:3. 10.1186/1475-2867-7-3 17391526 PMC1852544

[45] Saleiro D Platanias LC. Intersection of mTOR and STAT signaling in immunity. Trends Immunol. (2015) 36:21–9. 10.1016/j.it.2014.10.006 25592035 PMC4297313

[46] Corradetti MN Inoki K Guan KL. The stress-inducted proteins RTP801 and RTP801L are negative regulators of the mammalian target of rapamycin pathway. J Biol Chem. (2005) 280:9769–72. 10.1074/jbc.C400557200 15632201

[47] Armstrong JA Hart PD. Response of cultured macrophages to *Mycobacterium tuberculosis*, with observations on fusion of lysosomes with phagosomes. J Exp Med. (1971) 134:713–40. 10.1084/jem.134.3.713 15776571 PMC2139093

[48] Macgurn JA Cox JS. A genetic screen for *Mycobacterium tuberculosis* mutants defective for phagosome maturation arrest identifies components of the ESX-1 secretion system. Infect Immun. (2007) 75:2668–78. 10.1128/IAI.01872-06 17353284 PMC1932882

[49] Ramachandra L Noss E Boom WH Harding CV. Processing of *Mycobacterium tuberculosis* antigen 85B involves intraphagosomal formation of peptide-major histocompatibility complex II complexes and is inhibited by live bacilli that decrease phagosome maturation. J Exp Med. (2001) 194:1421–32. 10.1084/jem.194.10.1421 11714749 PMC2193679

[50] Watson RO Manzanillo PS Cox JS. Extracellular *M. tuberculosis* DNA targets bacteria for autophagy by activating the host DNA-sensing pathway. Cell. (2012) 150:803–15. 10.1016/j.cell.2012.06.040 22901810 PMC3708656

[51] Mehta M Rajmani RS Singh A. *Mycobacterium tuberculosis* WhiB3 responds to vacuolar pH-induced changes in mycothiol redox potential to modulate phagosomal maturation and virulence. J Biol Chem. (2016) 291:2888–903. 10.1074/jbc.M115.684597 26637353 PMC4742752

[52] Abu-Remaileh M Wyant GA Kim C Laqtom NN Abbasi M Chan SH. Lysosomal metabolomics reveals V-ATPase- and mTOR-dependent regulation of amino acid efflux from lysosomes. Science. (2017) 358:807–13. 10.1126/science.aan6298 29074583 PMC5704967

[53] Voskuil MI Bartek IL Visconti K Schoolnik GK. The response of *mycobacterium tuberculosis* to reactive oxygen and nitrogen species. Front Microbiol. (2011) 2:105. 10.3389/fmicb.2011.00105 21734908 PMC3119406

[54] Mahakalkar SM Nagrale D Gaur S Urade C Murhar B Turankar A. N-acetylcysteine as an add-on to directly observed therapy short-I therapy in fresh pulmonary tuberculosis patients: a randomized, placebo-controlled, double-blinded study. Perspect Clin Res. (2017) 8:132–6. 10.4103/2229-3485.210450 28828308 PMC5543764

[55] Garg SK Volpe E Palmieri G Mattei M Galati D Martino A. Sphingosine 1-phosphate induces antimicrobial activity both *in vitro* and *in vivo*. J Infect Dis. (2004) 189:2129–38. 10.1086/386286 15143482

[56] Thompson CR Iyer SS Melrose N Vanoosten R Johnson K Pitson SM. Sphingosine kinase 1 (SK1) is recruited to nascent phagosomes in human macrophages: inhibition of SK1 translocation by *Mycobacterium tuberculosis*. J Immunol. (2005) 174:3551–61. 10.4049/jimmunol.174.6.3551 15749892

[57] Sharma L Prakash H. Sphingolipids are dual specific drug targets for the management of pulmonary infections: perspective. Front Immunol. (2017) 8:378. 10.3389/fimmu.2017.00378 28400772 PMC5372786

[58] Belone Ade F Rosa PS Trombone AP Fachin LR Guidella CC Ura S. Genome-wide screening of mRNA expression in leprosy patients. Front Genet. (2015) 6:334. 10.3389/fgene.2015.00334 26635870 PMC4653304

[59] Guerreiro LT Robottom-Ferreira AB Ribeiro-Alves M Toledo-Pinto TG Rosa Brito T Rosa PS. Gene expression profiling specifies chemokine, mitochondrial and lipid metabolism signatures in leprosy. PLoS ONE. (2013) 8:e64748. 10.1371/journal.pone.0064748 23798993 PMC3683049

[60] Dupnik KM Bair TB Maia AO Amorim FM Costa MR Keesen TS. Transcriptional changes that characterize the immune reactions of leprosy. J Infect Dis. (2015) 211:1658–76. 10.1093/infdis/jiu612 25398459 PMC4425823

[61] Galagan JE Minch K Peterson M Lyubetskaya A Azizi E Sweet L. The Mycobacterium tuberculosis regulatory network and hypoxia. Nature. (2013) 499:178–83. 10.1038/nature12337 23823726 PMC4087036

[62] Marakalala MJ Raju RM Sharma K Zhang YJ Eugenin EA Prideaux B. Inflammatory signaling in human tuberculosis granulomas is spatially organized. Nat Med. (2016) 22:531–8. 10.1038/nm.4073 27043495 PMC4860068

[63] Cadena AM Fortune SM Flynn JL. Heterogeneity in tuberculosis. Nat Rev Immunol. (2017) 17:691–702. 10.1038/nri.2017.69 28736436 PMC6247113

[64] Esterhuyse MM Weiner J III Caron E Loxton AG Iannaccone M Wagman C. Epigenetics and proteomics join transcriptomics in the quest for tuberculosis biomarkers. MBio. (2015) 6:e01187–15. 10.1128/mBio.01187-15 26374119 PMC4600108

[65] Fletcher HA Snowden MA Landry B Rida W Satti I Harris SA T-cell activation is an immune correlate of risk in BCG vaccinated infants. Nat Commun. (2016) 7:11290 10.1038/ncomms11290 27068708 PMC4832066

[66] Wu TD Nacu S. Fast and SNP-tolerant detection of complex variants and splicing in short reads. Bioinformatics. (2010) 26:873–81. 10.1093/bioinformatics/btq057 20147302 PMC2844994

[67] Evans AM Dehaven CD Barrett T Mitchell M Milgram E. Integrated, nontargeted ultrahigh performance liquid chromatography/electrospray ionization tandem mass spectrometry platform for the identification and relative quantification of the small-molecule complement of biological systems. Anal Chem. (2009) 81:6656–67. 10.1021/ac901536h 19624122

[68] Dehaven CD Evans AM Dai H Lawton KA. Organization of GC/MS and LC/MS metabolomics data into chemical libraries. J Cheminform. (2010) 2:9. 10.1186/1758-2946-2-9 20955607 PMC2984397

[69] Sud M Fahy E Cotter D Azam K Vadivelu I Burant C. Metabolomics Workbench: an international repository for metabolomics data and metadata, metabolite standards, protocols, tutorials and training, and analysis tools. Nucleic Acids Res. (2016) 44:D463–70. 10.1093/nar/gkv1042 26467476 PMC4702780

[70] Weiner J III tmod: Feature Set Enrichment Analysis for Metabolomics and Transcriptomics. 0.40 ed Berlin (2018).

[71] R Core Team. R: A Language and Environment for Statistical Computing. Vienna: R Foundation for Statistical Computing (2016).

[72] Kuhn M Building predictive models in R using the caret package. J Stati Softw. (2008) 28:5 10.18637/jss.v028.i05

[73] Robin X Turck N Hainard A Tiberti N Lisacek F Sanchez JC. pROC: an open-source package for R and S+ to analyze and compare ROC curves. BMC Bioinformatics. (2011) 12:77. 10.1186/1471-2105-12-77 21414208 PMC3068975

[74] Wickham H ggplot2: Elegant Graphics for Data Analysis. New York, NY: Springer-Verlag (2009).

[75] Zeileis A Hothorn T Diagnostic checking in regression relationships. R News. (2002) 2:7–10.

[76] Durinck S Spellman PT Birney E Huber W. Mapping identifiers for the integration of genomic datasets with the R/Bioconductor package biomaRt. Nat Protoc. (2009) 4:1184–91. 10.1038/nprot.2009.97 19617889 PMC3159387

[77] Tenenbaum D KEGGREST: Client-side REST access to KEGG Seattle, WA (2016).

[78] Carlson M org.Hs.eg.db: Genome wide annotation for Human Seattle, WA (2016).

