# Supplementary figures and images for: Immunometabolic Signatures Predict Risk of Progression to Active Tuberculosis and Disease Outcome
Source: Front Immunol. 2019 Mar 22;10:527. doi: 10.3389/fimmu.2019.00527 (PMC6440524; doi:10.3389/fimmu.2019.00527)

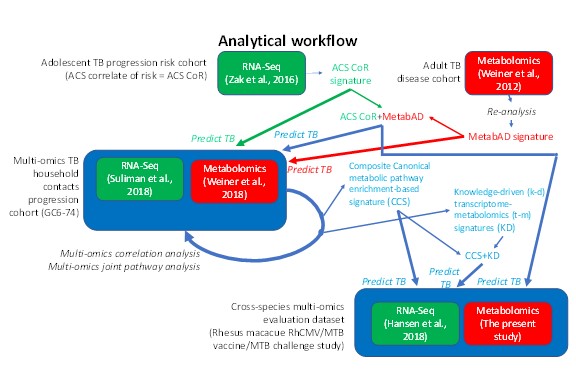

Supplement: Figure S1 — Overview of the multi-step analytical strategy employed to test whether integration of blood transcriptional profiling with serum metabolomic profiling can provide new understanding of disease processes and enable improved prediction of TB progression. ACS CoR, Adolescent Cohort Signature Correlate of Risk; MetabAD, Metabolomics Active Disease signature; CCS, Composite Canonical Signature; CCS+KD, Composite Canonical Signature plus Knowledge Driven pathways. [file Image_1.JPEG]

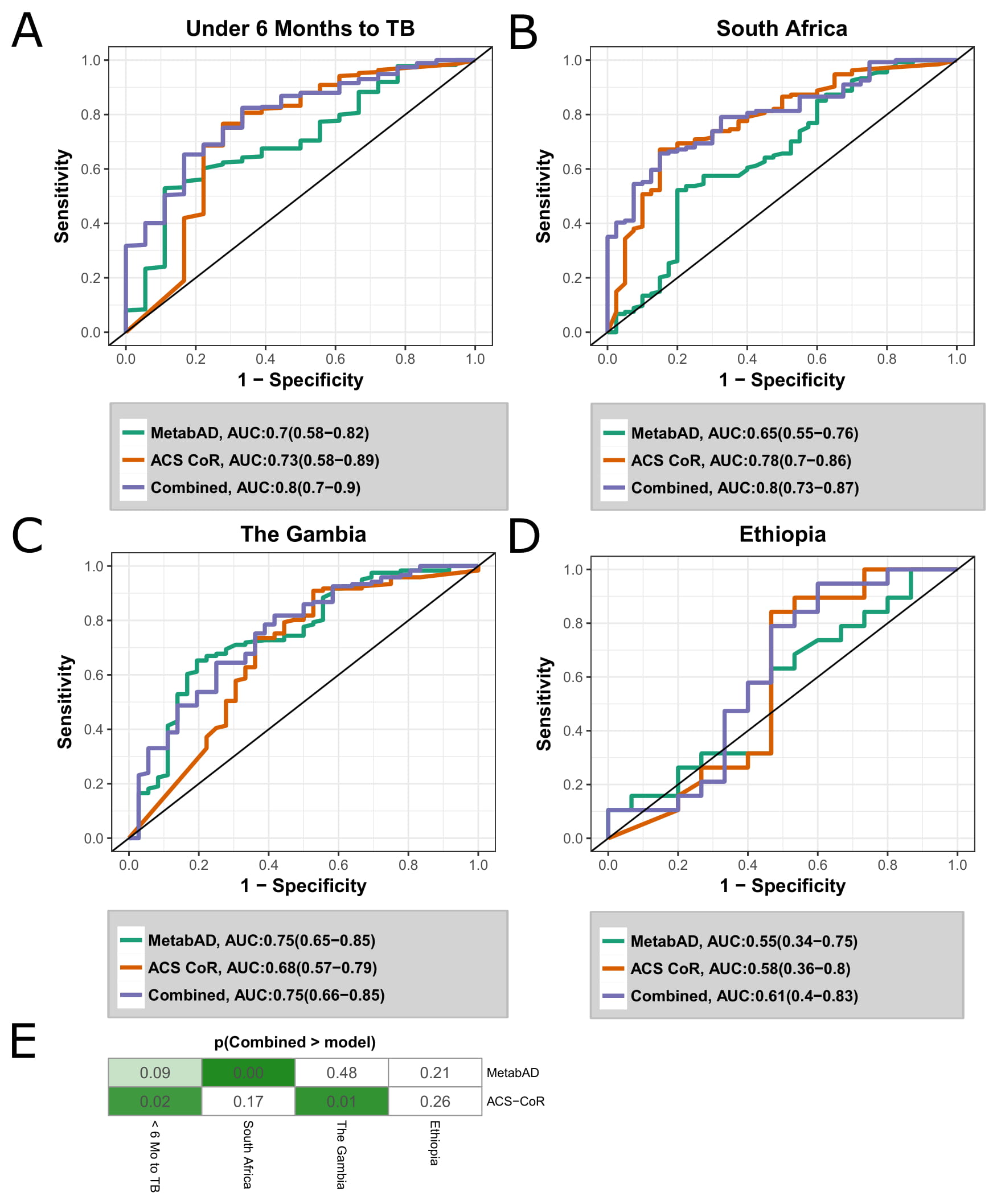

Supplement: Figure S2 — ROC curves of ACS-CoR, MetabAD, and combined ACS CoR + MetabAD signature predictions on subsets on the GC6-74 samples. (A) Progressors within 6 months of active TB vs. healthy controls. (B–D) predictions of specific samples from the South African, Gambian, and Ethiopian sites, respectively. (E) P-values (single-tailed Delong test) indicating whether the improvement in prediction for the ACS CoR+MetabAD model over either individual model is significant for each subset. [file Image_2.JPEG]

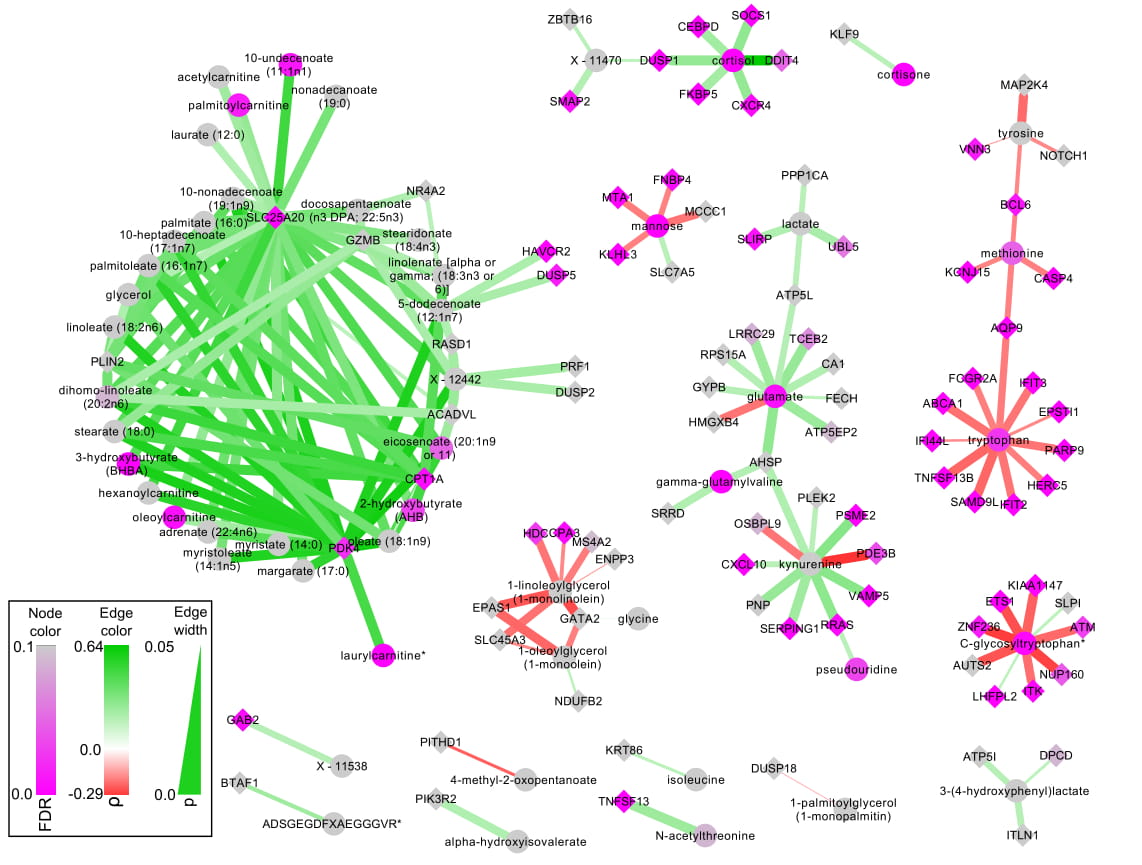

Supplement: Figure S3 — Network plot of all transcript/metabolite pairs previously identified as correlated in KORA F4 that are also significantly correlated in GC6-74 samples. Transcript nodes are shown as diamonds, metabolite nodes as circles, with significant correlations indicated by edges linking transcripts and metabolites. Positive correlations between metabolites and transcripts are shown as green edges and negative correlations as red. Darker shades indicate stronger correlations (legend shown bottom left). Transcripts and metabolites that showed significant association with TB progression were shaded in purple, with unassociated nodes shaded gray. Darker shades indicate more significant association, according to legend in bottom left. [file Image_3.JPEG]

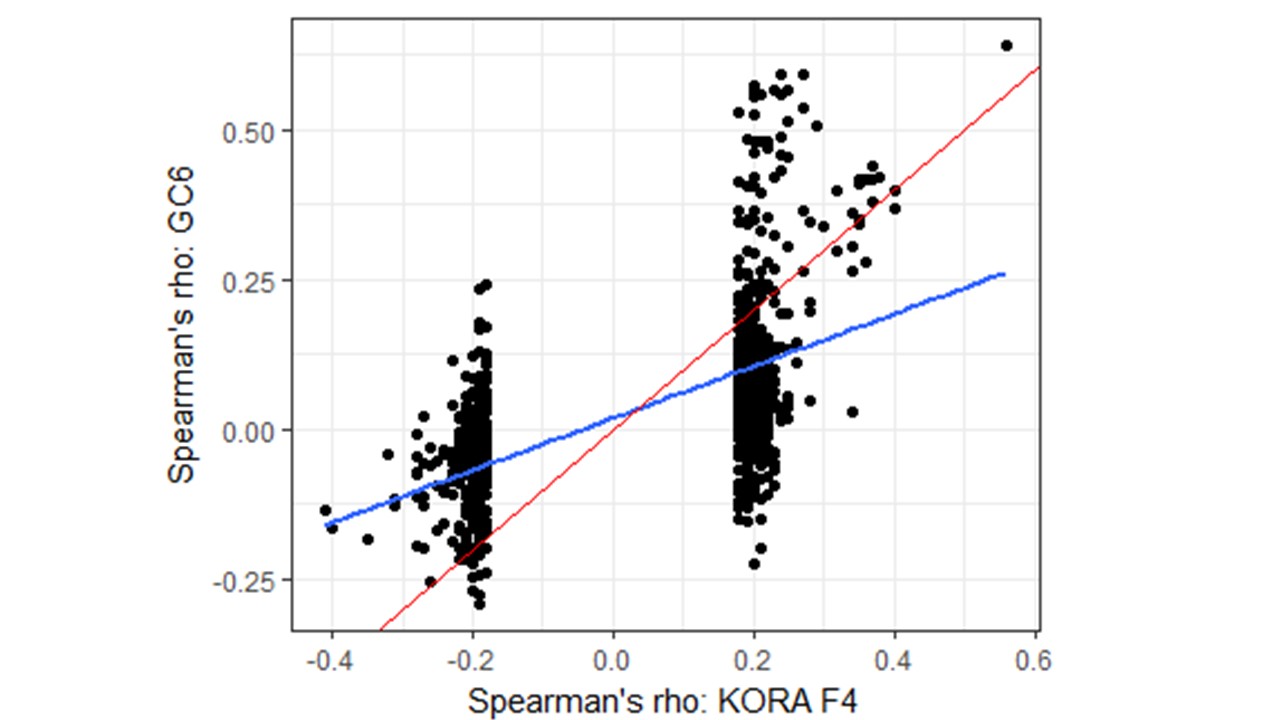

Supplement: Figure S4 — Concordance of correlations between the KORA F4 and GC6-74 transcript/metabolite pairs. Each point represents a single (t-m) pair significantly correlated in the KORA F4 cohort. The x-axis shows Spearman correlation coefficients for (t-m) pairs from the KORA F4 cohort, and the y-axis shows the equivalent correlations in GC6-74. The red line represents x=y, perfect concordance, and the blue line is the linear best fit. [file Image_4.JPEG]

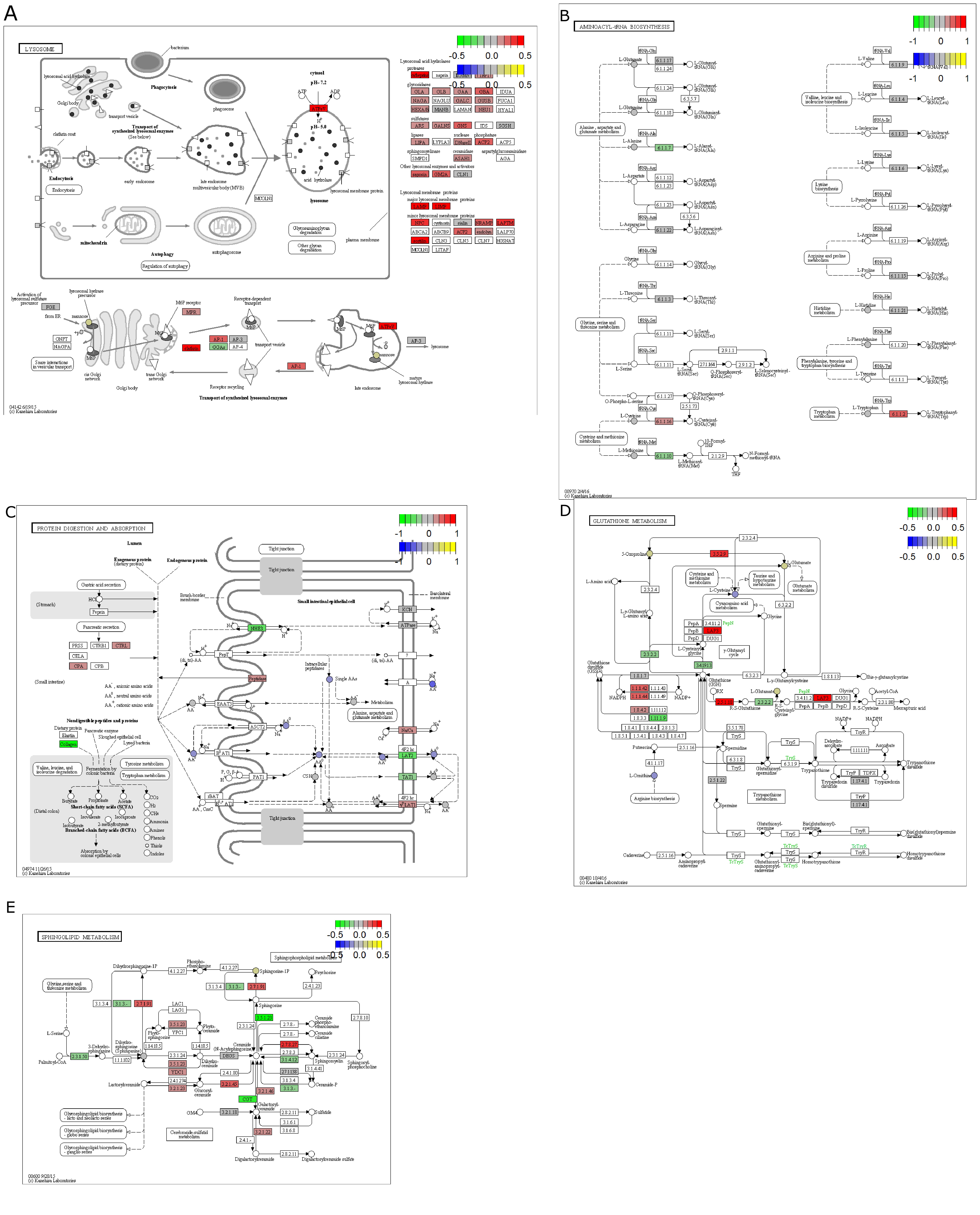

Supplement: Figure S5 — KEGG pathway maps for each of the significant KEGG pathways shown in Table 3. (A–E) Maps for lysosome, aminoacyl-tRNA synthesis, protein digestion and absorption, glutathione metabolism, and sphigolipid metabolism respectively. Significantly up- and down-regulated transcripts are highlighted in red and green, respectively, and significantly up- and down-regulated metabolites are shown in blue and yellow, respectively. [file Image_5.JPEG]

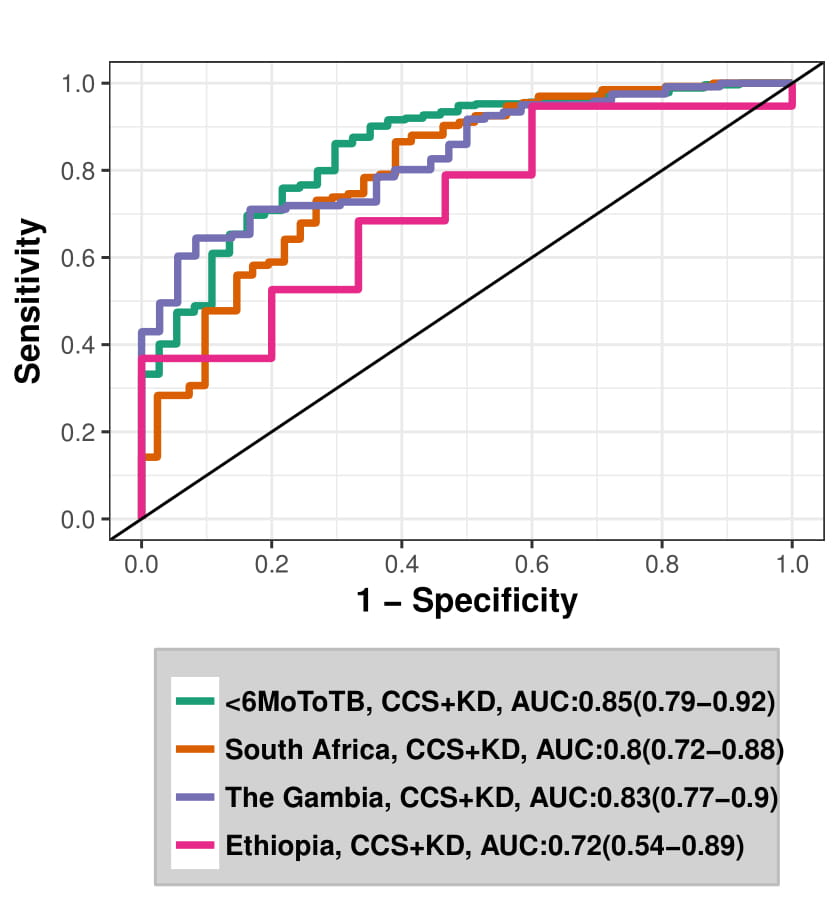

Supplement: Figure S6 — ROC curves for the CCS+KD classifier on subsets of the GC6-74 cohort. Signature performance is shown for progressors within 6 months of active disease, and for each individual study site. [file Image_6.JPEG]

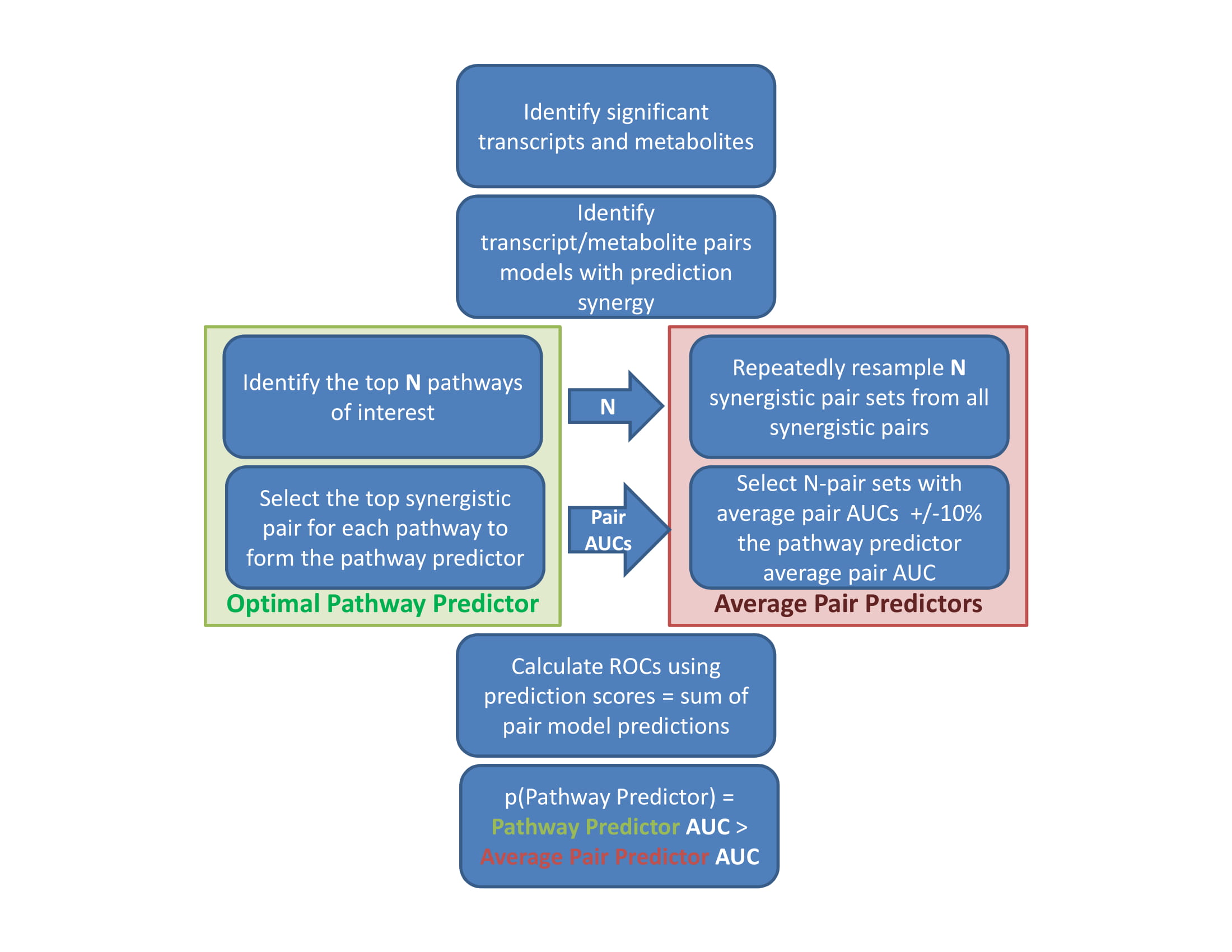

Supplement: Figure S7 — Flow diagram illustrating the resampling procedure used to compare performance of the pathway-derived signature to randomly constructed signatures containing the same number of (t-m) pairs with similar predictive performance. [file Image_7.JPEG]

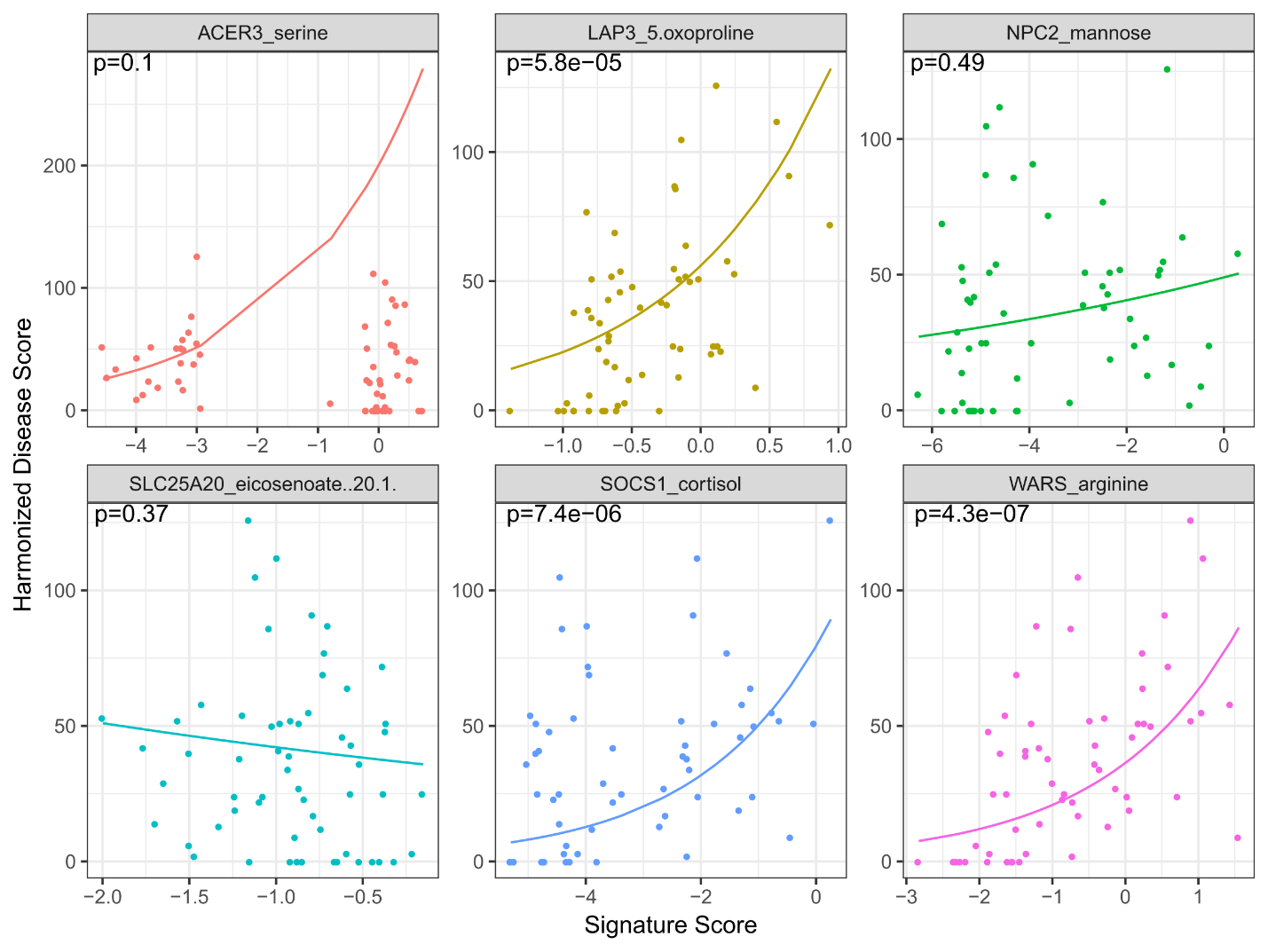

Supplement: Figure S8 — Scatter plots of individual pairs from the CCS+KD classifier compared to harmonized disease scores from two independent RM vaccine trial studies measured 28 days post-challenge with M.tb. Solid lines indicate the best-fit Poisson model, and p-values shown indicate Poisson p-values for the association between individual pair score 28 days post-challenge and harmonized disease score. [file Image_8.TIF]
